# Autoinducer-2 enhances the defense of *Vibrio furnissii* against oxidative stress and DNA damage by modulation of c-di-GMP signaling via a two-component system

**DOI:** 10.1128/mbio.02922-24

**Published:** 2025-01-16

**Authors:** Heng Zhang, Wenjin Zhao, Wenguang Yang, Huimin Zhang, Xinyu Qian, Kai Sun, Qiao Yang, Xihui Shen, Lei Zhang

**Affiliations:** 1 State Key Laboratory for Crop Stress Resistance and High-Efficiency Production, Shaanxi Key Laboratory of Agricultural and Environmental Microbiology, College of Life Sciences, Northwest A&F University, Yangling, Shaanxi, China; 2 ABI Group, Phycosphere Microbiology Laboratory, Zhejiang Ocean University, Zhoushan, China; Hebrew University of Jerusalem Robert H Smith Faculty of Agriculture Food and Environment, Rehovot, Israel; Indiana University Bloomington, Bloomington, Indiana, USA

**Keywords:** autoinducer-2, two-component system, c-di-GMP, oxidative stress, DNA damage

## Abstract

As a universal language across the bacterial kingdom, the quorum sensing signal autoinducer-2 (AI-2) can coordinate many bacterial group behaviors. However, unknown AI-2 receptors in bacteria may be more than what has been discovered so far, and there are still many unknown functions for this signal waiting to be explored. Here, we have identified a membrane-bound histidine kinase of the pathogenic bacterium *Vibrio furnissii*, AsrK, as a receptor that specifically detects AI-2 under low boron conditions. In contrast with another well-known AI-2 receptor LuxP that recognizes the borated form of AI-2, AsrK is found to show higher affinity with AI-2 under borate-depleted conditions, and thus boron has a negative effect on AI-2 sensing by AsrK in regulation of the biofilm and motility phenotypes. AI-2 binds to the extracytoplasmic dCache_1 domain of AsrK to inhibit its autokinase activity, thus decreasing the phosphorylation level of its cognate response regulator AsrR and activating the phosphodiesterase activity of AsrR to degrade the cellular second messenger cyclic di-GMP (c-di-GMP). AI-2 perception by the AsrK-AsrR system remarkably reduces intracellular c-di-GMP levels and enhances tolerance of *V. furnissii* to oxidative stress and DNA damage by upregulating the transcription of universal stress proteins including UspA1, UspA2, and UspE. Our study reveals a previously unrecognized mechanism for AI-2 detection in bacteria and also provides new insights into the important role of AI-2 in bacterial defense against oxidative stress and DNA damage.

**IMPORTANCE:**

The QS signal AI-2 is widely synthesized in bacteria and has been implicated in the regulation of numerous bacterial group behaviors. However, in contrast to the wide distribution of this signal, its receptors have only been found in a small number of bacterial species, and the underlying mechanisms for the detection of and response to AI-2 remain elusive in most bacteria. It is worth noting that the periplasmic protein LuxP is the uniquely identified receptor for AI-2 in *Vibrio* spp. Here, we identify a second type of AI-2 receptor, a membrane-bound histidine kinase with a periplasmic dCache_1 sensory domain, in a member of the genus *Vibrio*, and thus show that AI-2 enhances the defense of *V. furnissii* against oxidative stress and DNA damage by modulation of c-di-GMP signaling via the AsrK-AsrR two-component system. Our results reveal a previously unrecognized AI-2 sensing mechanism and expand our understanding of the physiological roles of AI-2 in bacteria.

## INTRODUCTION

Quorum sensing (QS) is a cell-cell communication mechanism that controls bacterial group behaviors in a cell density-dependent manner (1, 2). QS relies on the production, release, accumulation, and detection of signal molecules called autoinducers (3–5). Although most autoinducers including acyl-homoserine lactones (AHLs) produced by Gram-negative bacteria and autoinducing peptides produced by Gram-positive bacteria are used for intraspecies communication (6, 7), autoinducer-2 (AI-2) that is produced by many Gram-negative and Gram-positive bacteria could participate in both intra- and interspecies communication (8–10).

The AI-2 precursor 4,5-dihydroxy-2,3-pentanedione (DPD) is generally synthesized by LuxS from S-ribosylhomocysteine, and then DPD cyclizes spontaneously into different isomers including active AI-2 forms (10–12). To date, two active AI-2 forms have been identified, including the borated form called S-2-methyl-2,3,3,4-tetrahydroxytetrahydrofuran-borate (S-THMF-borate) and the non-borated form called R-2-methyl-2,3,3,4-tetrahydroxytetrahydrofuran (R-THMF) (11, 12). Correspondingly, two classes of periplasmic AI-2 receptors, LuxP found only in *Vibrio* spp. and LsrB present in enteric bacteria and members of several other families, exclusively bind S-THMF-borate and R-THMF, respectively (11–16). Recently, our studies identified two other types of membrane-bound AI-2 receptors with periplasmic sensory domains, including dCache_1 domain-containing receptors widely distributed in bacteria (10) and GAPES1 domain-containing receptors found in enteric bacteria (17). Nevertheless, which AI-2 form was detected by dCache_1 domain- and GAPES1 domain-containing receptors remain elusive, and the underlying mechanisms for the detection of and response to AI-2 are still largely unknown.

Cyclic di-GMP (c-di-GMP) is recognized as a near-ubiquitous bacterial second messenger that controls a wide range of biological processes, including biofilm formation, virulence, motility, cell cycle progression, and stress adaptation (17–21). c-di-GMP is synthesized from two molecules of GTP by diguanylate cyclases (DGCs) that have a GGDEF domain, and is degraded into 5′-phosphoguanylyl-(3′,5′)-guanosine (pGpG) or GMP by c-di-GMP-specific phosphodiesterases (PDEs) containing either an EAL or an HD-GYP domain (17–19, 21, 22). While bacterial genomes usually encode several to dozens of DGCs and PDEs, many of these c-di-GMP-metabolizing proteins have periplasmic or cytoplasmic sensory domains linked to the catalytic domains (10, 17, 23–25). Cellular c-di-GMP levels are modulated by changes in the expression or activity of DGCs or PDEs in response to internal and external signals (23–29). AI-2 has been shown to induce c-di-GMP synthesis via directly targeting a GAPES1 domain-containing DGC in *Salmonella enterica* serovar Typhimurium (17). Signal perception of AI-2 in the periplasm by LuxP converts the activity of the transmembrane histidine kinase (HK) LuxQ from kinase to phosphatase and leads to dephosphorylation of the response regulator (RR) LuxO, ultimately regulating the transcription of multiple c-di-GMP-metabolizing enzymes and decreasing c-di-GMP levels in *Vibrio cholerae* (30).

In this work, we show that a transmembrane sensor HK of the pathogenic bacterium *V. furnissii*, AsrK, specifically binds AI-2 under low boron conditions to decrease its autokinase activity. AI-2 perception by AsrK decreases the phosphorylation level of the cognate RR AsrR and activates its PDE activity in degrading the second messenger c-di-GMP. This signaling process promotes bacterial tolerance to oxidative stress and DNA damage via upregulating the transcription of several universal stress proteins. Our study reveals a previously unrecognized regulatory connection between the QS signal AI-2 and c-di-GMP and significantly extends our knowledge of the function of AI-2 in stress resistance.

## RESULTS

### AI-2 regulates biofilm formation and swimming motility in *V. furnissii* without the *luxP* gene

**Fig 1:**
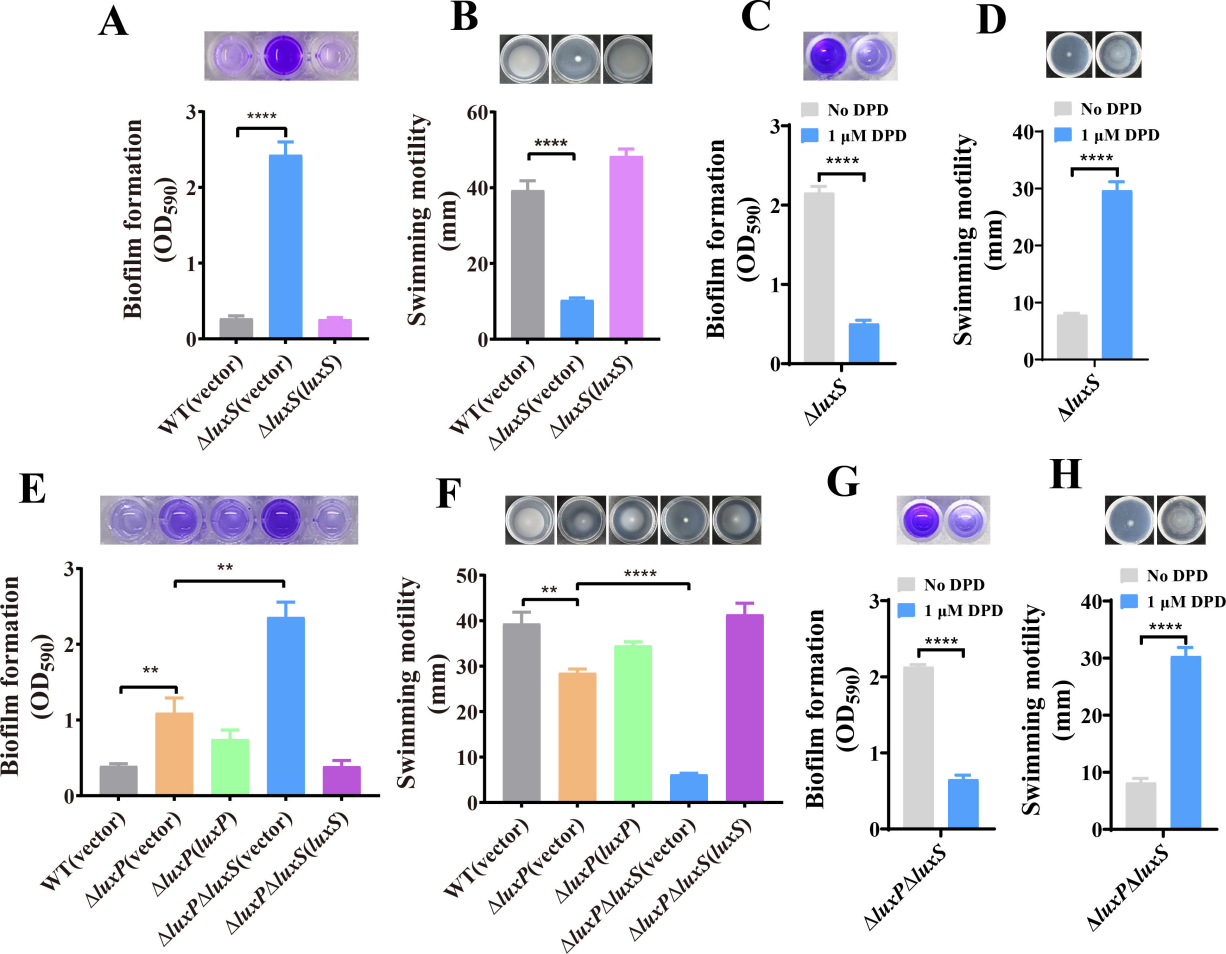
AI-2 regulates biofilm formation and swimming motility of *V. furnissii* independent of LuxP. (**A**) Deletion of the *luxS* gene increases the biofilm formation of *V. furnissii*. The biofilm was stained with 0.1% crystal violet and absorbance was measured at 590 nm for quantification. (**B**) Deletion of *luxS* reduces the swimming motility of *V. furnissii*. The swimming motility of the indicated strains was tested in the semisolid agar medium, and the diameter of motility halos was measured. (**C**) Biofilm formation of Δ*luxS* is inhibited by the exogenous addition of 1 µM DPD. (**D**) The swimming motility of Δ*luxS* is enhanced by the exogenous addition of 1 µM DPD. (**E and F**) Deletion of *luxS* in the Δ*luxP* mutant also significantly increases biofilm formation (**E**) and reduces swimming motility (**F**). (**G and H**) AI-2 signal supplementation (1 µM DPD) reduces biofilm formation (**G**) and increases swimming motility (**H**) of Δ*luxP*Δ*luxS*. In panels A–H, all data represent mean ± s.d. of three independent experiments, each experiment having three technical replicates. Statistical significance was evaluated using Student’s *t*-test. **P < 0.01; ****P < 0.0001.

Two different types of AI-2 receptors, the periplasmic protein LsrB that interacts with the membrane components of an ABC transport system called Lsr involved in AI-2 uptake and the transmembrane GAPES1 domain-containing DGC YeaJ involved in c-di-GMP synthesis, have been identified in enteric bacteria such as *S*. Typhimurium (12, 17). However, the periplasmic protein LuxP that functions in conjunction with the two-component HK LuxQ is the only identified receptor for AI-2 in *Vibrio* spp. (11). It is unknown whether additional AI-2 receptors exist in members of the genus *Vibrio*. We found that the deletion of *luxS* led to over ninefold enhanced biofilm formation but decreased swimming motility by 74% in *V. furnissii* (Fig. 1A and B). Moreover, the addition of 1 µM DPD led to significantly reduced biofilm formation and increased swimming motility in the Δ*luxS* mutant (Fig. 1C and D), indicating that AI-2 regulates biofilm formation and swimming motility in *V. furnissii*. Deletion of the *luxP* gene in *V. furnissii* also significantly increased biofilm formation and reduced swimming motility, but to a lesser extent as compared to the *luxS* deletion (Fig. 1E and F). Furthermore, the mutant Δ*luxP*Δ*luxS* showed significantly enhanced biofilm formation and decreased swimming motility compared with the Δ*luxP* mutant, and such changes were completely restored by complementation with a plasmid encoding *luxS* (Fig. 1E and F). In agreement, the addition of 1 µM DPD resulted in significantly reduced biofilm formation and increased swimming motility in Δ*luxP*Δ*luxS* (Fig. 1G and H). Our results indicate that AI-2 could play a role in the motile-sessile transition independent of its receptor LuxP in *V. furnissii,* thus suggesting the presence of other AI-2 receptors besides LuxP in this bacterium.

### AI-2 directly binds to the periplasmic dCache_1 domain of AsrK to inhibit its autokinase activity

**Fig 2:**
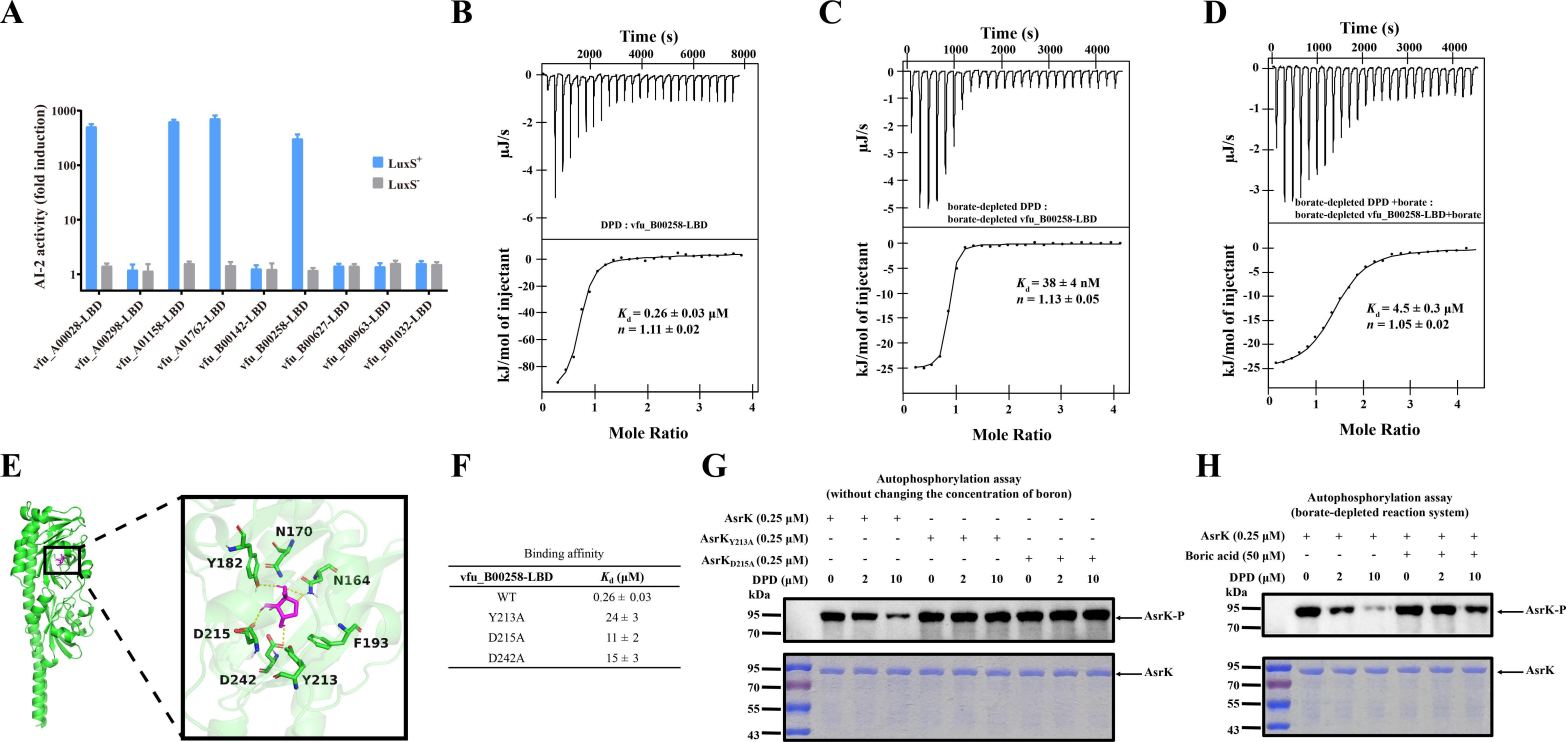
The HK AsrK, encoded by the gene vfu_B00258, is a novel receptor for AI-2. (**A**) The LBDs of vfu_A00028, vfu_A01158, vfu_A01762, and vfu_B00258 are capable of retaining AI-2. Bioluminescence in *V. Halveyi* MM32 was measured following the addition of ligands released from purified proteins expressed in *E. coli* BL21 (DE3) with or without *luxS*. AI-2 activity is reported as fold induction relative to light production induced by a buffer control. (**B**) vfu_B00258-LBD interacts with DPD with high affinity. ITC data and plots of injected heat for injections of DPD solution (150 µM) into the sample cell containing 10 µM vfu_B00258-LBD are shown in the upper and lower plots, respectively. (**C and D**) Higher binding affinity of DPD to vfu_B00258-LBD is detected under borate-depleted conditions. DPD was diluted in a borate-depleted buffer to 30 µM, and the vfu_B00258-LBD protein was dialyzed against and diluted in the same buffer to 2 µM before use. The borate-depleted DPD (**C**) or the borate-depleted DPD supplemented with 150 µM boric acid (**D**) was titrated with the borate-depleted vfu_B00258-LBD (**C**) or the borate-depleted vfu_B00258-LBD supplemented with 150 µM boric acid (**D**). (**E**) Predicted binding mode of R-THMF in the membrane-distal pocket of vfu_B00258-LBD. (**F**) ITC analysis of the binding of DPD to the vfu_B00258-LBD mutants without changing the concentration of boron. (**G**) AI-2 decreases the autokinase activity of AsrK. AsrK, AsrK_Y213A_, and AsrK_D215A_ were incubated with ATP-γ-S in the presence of different concentrations (0, 2, and 10 µM) of DPD before autophosphorylation detection. (**H**) Boric acid greatly weakens the inhibition effect of DPD on the autokinase activity of AsrK. In a borate-depleted reaction system, the borate-depleted AsrK was incubated with ATP-γ-S in the presence of different concentrations (0, 2, and 10 µM) of borate-depleted DPD with or without added 50 µM boric acid. In panels G and H, the thiophospholyation site on the substrates was alkylated by p-nitrobenzyl mesylate and then immunoblotted with the antibody against thiophosphate ester (top). Gel stained with Coomassie brilliant blue to check protein amounts (bottom). In panels A–D and F, values were mean  ±  s.d. of three independent experiments. In panels B–D, G, and H, the ITC data, gels, and blots shown are representatives of three independent experiments with similar results.

Our recent study has shown that dCache_1 domain-containing AI-2 receptors are widely present in bacteria (10). Thus, we investigated whether this type of AI-2 receptor is present in *V. furnissii*. Protein domain annotations of 4462 proteins encoded by its genome (CP002377 and CP002378) by hmmscan program in HMMER (https://www.ebi.ac.uk/Tools/hmmer/search/hmmscan) showed that nine transmembrane proteins, including seven methyl-accepting chemotaxis proteins (MCPs), one putative DGC and one HK, have the dCache_1 domain model (PF02743) as the best hit. We thus examined whether these periplasmic dCache_1 domains can bind AI-2. In the *Vibrio harveyi* MM32 reporter assays, AI-2-binding activity was observed for the dCache_1 domains of three MCPs vfu_A00028, vfu_A01158, and vfu_A01762, and the HK vfu_B00258, but not for the dCache_1 domains of the other five proteins (Fig. 2A). While two MCPs PctA and TlpQ of *Pseudomonas aeruginosa* sense AI-2 via their dCache_1 domains to induce chemotaxis (10), the three MCPs of *V. furnissii* should mediate chemotaxis of this bacterium toward AI-2. However, it remains unclear whether and how the HK vfu_B00258 senses AI-2 to regulate important functions in *V. furnissii*.

We further determined the binding affinity between AI-2 and the dCache_1 domain of vfu_B00258 by isothermal titration calorimetry (ITC) and found that the disassociation constant (*K*_*d*_) value between them is 0.26 ± 0.03 µM (Fig. 2B), which is comparable to the *K*_*d*_ values of AI-2 for other well-established AI-2 receptors (14, 31). Whereas the *Vibrio* AI-2 receptor LuxP has been shown to bind the borated AI-2 form S-THMF-borate (11), the periplasmic ligand-binding domains (LBDs) of PctA and TlpQ were suggested to recognize the non-borated AI-2 form R-THMF (10). Given that boron is known to shift the equilibrium of AI-2 molecules toward the S-THMF-borate form (12), we investigated the role of boron in the binding of AI-2 to vfu_B00258-LBD. When DPD diluted in a borate-depleted buffer was titrated into vfu_B00258-LBD in a borate-depleted buffer, we detected a binding afﬁnity (*K*_*d*_) of 38 ± 4 nM (Fig. 2C). Moreover, the addition of 150 µM boric acid in both of the borate-depleted DPD and protein solutions led to a 118-fold decrease in binding afﬁnity (*K*_*d*_ = 4.5 ± 0.3 µM) (Fig. 2D). These results suggest that the non-borated form of AI-2 is the preferred ligand for vfu_B00258-LBD. Then, we predicted the 3D structure of the dCache_1 domain of vfu_B00258 by Alphafold2 and performed a docking simulation by AutoDock Vina 1.1.2 to analyze the interaction between vfu_B00258-LBD and the non-borated AI-2 form R-THMF. The best conformation of R-THMF bound to vfu_B00258-LBD (Fig. 2E) has the lowest docking score of −5.9 kcal mol^−1^, which is comparable to that (−5.7 kcal mol^−1^) of AI-2-TlpQ-LBD docking (10). This conformation suggests that AI-2 makes close contact with N164, N170, Y182, F193, Y213, D215, and D242 in the membrane-distal pocket. In support of this binding model, alignment of the dCache_1 domain from vfu_B00258 and PctA showed that vfu_B00258-LBD contains three residues Y213, D215, and D242 corresponding to Y144, D146, and D173 in PctA-LBD that are important for AI-2 binding (10) (Fig. S1). Furthermore, alanine substitution mutations in Y213, D215, or D242 resulted in marked a reduction in AI-2-binding affinity for vfu_B00258-LBD (Fig. 2F; Fig. S2). These results indicate that AI-2 is a high-affinity ligand for vfu_B00258-LBD, and further suggest that vfu_B00258-LBD senses the non-borated form of AI-2 via the pocket within its membrane-distal modules.

We then determined whether AI-2 affects the autokinase activity of vfu_B00258. *In vitro* kinase assay showed that 2 µM DPD slightly decreases while 10 µM DPD substantially decreases the autophosphorylation level of vfu_B00258 (Fig. 2G). 2 µM DPD decreased the kinase activity of 0.25 µM vfu_B00258 so little, which does not quite match the ITC data that showed that the *K*_*d*_ of vfu_B00258 for AI-2 is sub-micromolar (Fig. 2B). We noticed that the reaction buffer used in the kinase assay was stored in a glass bottle, while the Tris buffer used for ITC analysis was stored in a plastic bottle. We thus speculated that the reaction buffer stored in a glass bottle probably contains a relatively high concentration of boron that weakens the effect of DPD on vfu_B00258. In contrast to wild-type vfu_B00258, the addition of 10 µM DPD did not cause a recognizable decrease in the autophosphorylation level of the point mutants vfu_B00258_Y213A_ and vfu_B00258_D215A_ (Fig. 2G). These results indicate that vfu_B00258 is an AI-2 sensing receptor kinase, thus named AsrK, and AI-2 binds to the dCache_1 domain of AsrK to inhibit its autokinase activity. In a borate-depleted reaction system, 2 and 10 µM borate-depleted DPD drastically decreased the autophosphorylation level of AsrK, while the addition of 50 µM boric acid in the system greatly weakened the inhibition effect of DPD on the autokinase activity of AsrK (Fig. 2H). These results further suggest that AsrK senses the non-borated form of AI-2 to inhibit its autokinase activity.

### The non-borated form of AI-2 regulates biofilm formation and swimming motility in *V. furnissii* via AsrK

**Fig 3:**
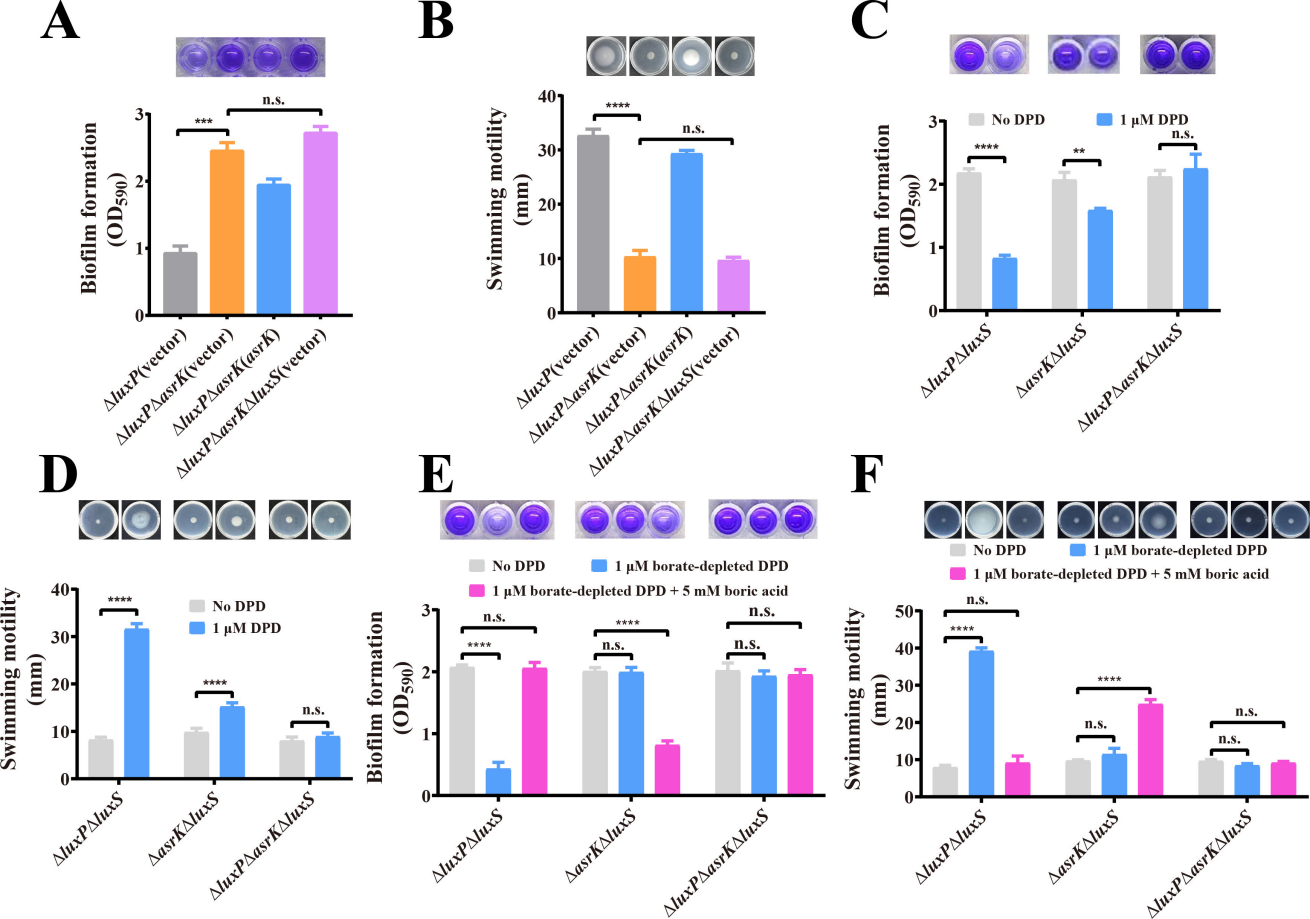
The non-borated form of AI-2 regulates biofilm formation and swimming motility in *V. furnissii via* AsrK. (**A and B**) Deletion of *asrK* in Δ*luxP* significantly increases biofilm formation (**A**) and reduces swimming motility (**B**), but deletion of *luxS* in Δ*luxP*Δ*asrK* leads to no significant changes in these phenotypes. (**C and D**) AI-2 signal supplementation significantly reduces biofilm formation (**C**) and increases swimming motility (**D**) in Δ*luxP*Δ*luxS* and Δ*asrK*Δ*luxS* but leads to no significant changes in these phenotypes in Δ*luxP*Δ*asrK*Δ*luxS*. (**E and F**) Boric acid inhibits the effects of borate-depleted DPD on the biofilm (**E**) and motility (**F**) phenotypes of Δ*luxP*Δ*luxS* but enhances the effects of borate-depleted DPD on these phenotypes of Δ*asrK*Δ*luxS* in borate-depleted media. In panels A–F, statistical analyses were carried out by results from three independent experiments, each experiment having three technical replicates. Data are mean ± s.d., and Student’s *t*-test was used for these analyses. n.s., not significant; **P < 0.01; ***P < 0.001; ****P < 0.0001.

We then determined whether AI-2 regulates the biofilm and motility phenotypes of *V. furnissii* via AsrK. The biofilm formation ability of Δ*luxP*Δ*asrK* was significantly increased, and the swimming ability was significantly decreased compared to Δ*luxP*, whereas deletion of *luxS* in Δ*luxP*Δ*asrK* did not lead to significant changes in these phenotypes (Fig. 3A and B). Moreover, the addition of 1 µM DPD significantly reduced biofilm formation and increased swimming motility in Δ*luxP*Δ*luxS,* but had no effect on the biofilm and motility phenotypes of Δ*luxP*Δ*asrK*Δ*luxS* (Fig. 3C and D). These results indicate that AI-2 regulates biofilm formation and swimming motility via AsrK in the absence of LuxP. Besides, the addition of 1 µM DPD significantly reduced biofilm formation and increased swimming motility in Δ*asrK*Δ*luxS* (Fig. 3C and D), suggesting that AI-2 detection by LuxP can also modulate the biofilm and motility phenotypes in *V. furnissii*. In borate-depleted media, the addition of 1 µM DPD diluted in borate-depleted water significantly reduced biofilm formation and increased swimming motility in Δ*luxP*Δ*luxS,* while the effects of DPD on the biofilm and motility phenotypes of this strain were completely abrogated when 5 mM boric acid was added to the borate-depleted media (Fig. 3E and F), further supporting that AsrK senses the non-borated form of AI-2. By contrast, the addition of borate-depleted DPD had no effect on the biofilm and motility phenotypes of Δ*asrK*Δ*luxS*, whereas the addition of boric acid together with borate-depleted DPD drastically changed its biofilm and motility phenotypes (Fig. 3E and F), which is consistent with the previous finding that LuxP recognizes the borated form of AI-2 (11). Nevertheless, the addition of borate-depleted DPD with or without boric acid had no effect on the biofilm and motility phenotypes of Δ*luxP*Δ*asrK*Δ*luxS* in the borate-depleted media (Fig. 3E and F), further indicating that AI-2 regulates the biofilm and motility phenotypes of *V. furnissii* via both AsrK and LuxP. Together, these results indicate that boron has a positive effect on AI-2 sensing by LuxP in the regulation of the biofilm and motility phenotypes, but has a negative effect on AI-2 sensing by AsrK in the regulation of these phenotypes.

### AI-2 regulates intracellular c-di-GMP levels via AsrK-AsrR two-component system

**Fig 4:**
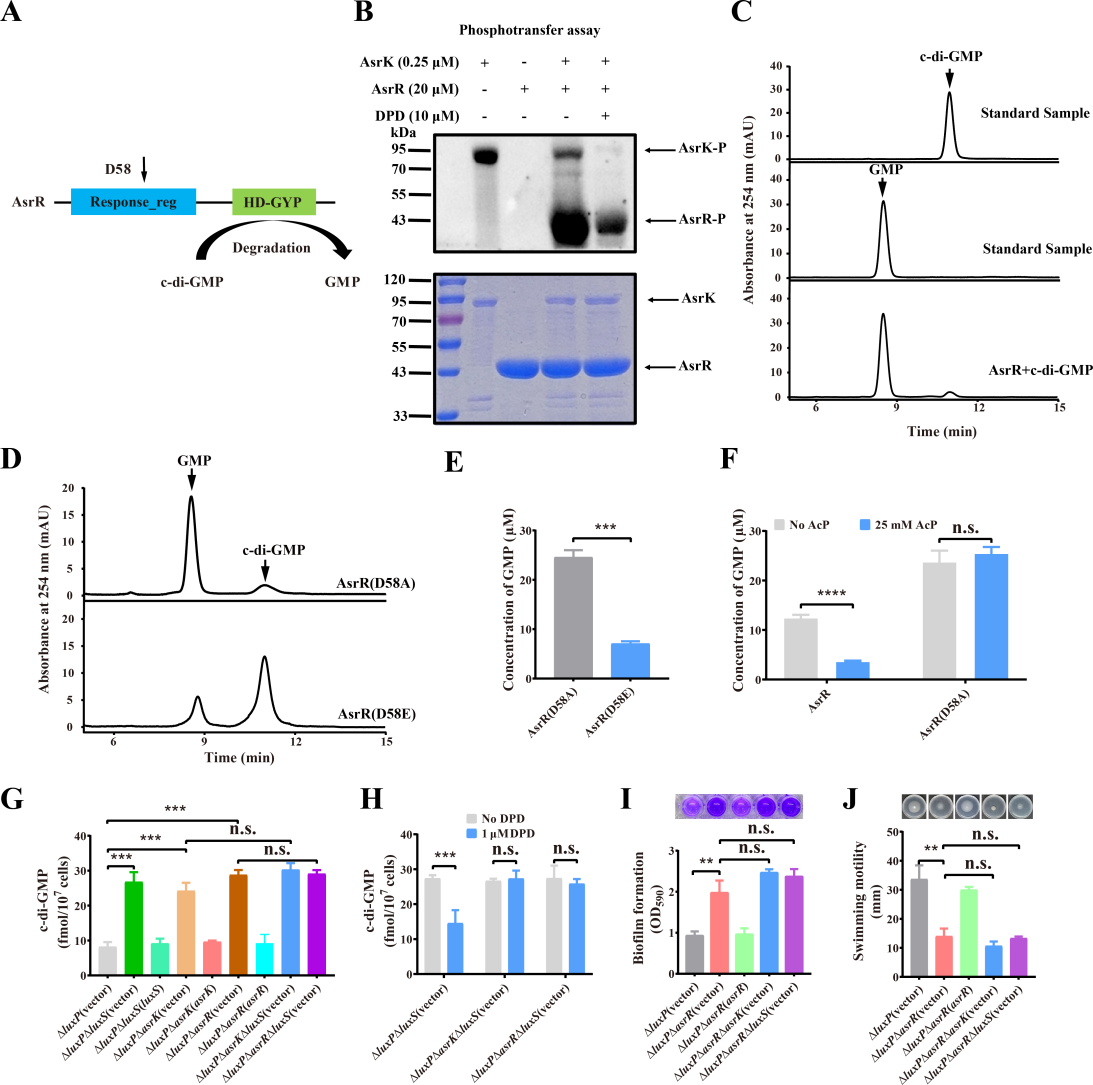
AI-2 decreases cellular c-di-GMP levels via the AsrK-AsrR TCS. (**A**) Schematic illustrating the predicted domain organization of AsrR. Domain names come from the pfam database. The conserved phosphorylation sites are labeled above the Response_reg domain. (**B**) The addition of DPD remarkably decreases phosphorylation levels of both AsrK and AsrR. AsrK and DPD were incubated at 30℃ in a reaction solution containing ATP-γ-S for 5 min, and then the AsrR protein was added to the reaction mixture for another 5 min before alkylation of the thiophospholyation site and detection with anti-thiophosphate ester antibody. (**C**) AsrR can degrade c-di-GMP. AsrR was incubated with c-di-GMP, and the reaction products were analyzed by HPLC. (**D**) Dephosphorylated AsrR has a higher PDE activity than phosphorylated AsrR in degrading c-di-GMP. (**E**) Quantification of the levels of GMP produced in panel D based on the standard curve established using known concentrations of GMP. (**F**) Acetyl phosphate (AcP) treatment significantly reduces the PDE activity of wild-type AsrR but not AsrR_D58A_. Purified AsrR and AsrR_D58A_ were pretreated by 25 mM AcP for 30 min at 30°C before incubation with c-di-GMP for another 30 min, and the levels of synthesized GMP were determined as described in panel E. (**G**) Deletion of *luxS, asrK* or *asrR* in the Δ*luxP* mutant leads to elevated intracellular c-di-GMP levels. (**H**) AI-2 signal supplementation significantly decreases cellular c-di-GMP concentration in Δ*luxP*Δ*luxS,* but not in Δ*luxP*Δ*asrK*Δ*luxS* or Δ*luxP*Δ*asrR*Δ*luxS*. (**I and J**) Deletion of *asrR* in Δ*luxP* significantly increases biofilm formation (**I**) and reduces swimming motility (**J**), but deletion of *luxS* or *asrK* in Δ*luxP*Δ*asrR* leads to no significant changes in these phenotypes. In B–D, the data shown are representatives of three independent experiments with similar results. In panels E–J, data are mean ± s.d. of three independent experiments. Student’s *t*-test was used for all statistical analyses. n.s., not significant; **P < 0.01; ***P < 0.001; ****P < 0.0001.

In the genome of *V. furnissii*, *asrK* is adjacent to *vfu_B00259*, which contains a Response_reg receiver domain and an HD-GYP output domain and thus is thought to encode the cognate RR of AsrK, named AsrR (Fig. 4A). We first purified His_6_-tagged AsrR and examined whether AsrK phosphorylates AsrR. *In vitro* phosphotransfer assay showed that AsrK directly phosphorylates AsrR (Fig. 4B), suggesting that AsrK-AsrR is a two-component system (TCS). Furthermore, the addition of 10 µM DPD remarkably decreased the phosphorylation of both AsrK and AsrR (Fig. 4B), indicating that the reduced autophosphorylation level of AsrK by AI-2 results in the reduced phosphorylation level of AsrR. Meanwhile, we examined whether AsrR has PDE activity. *In vitro* reaction of His_6_-tagged AsrR with c-di-GMP followed by HPLC analysis verified that AsrR can hydrolyze c-di-GMP into GMP (Fig. 4C). To investigate whether the phosphorylation level of AsrR affects its PDE activity in degradation of c-di-GMP, the predicted phosphorylation site D58 of AsrR was mutated to alanine or glutamic acid to result in the constitutive dephosphorylation and constitutive phosphorylation of AsrR, respectively. The *in vitro* PDE activity assay showed that AsrR_D58A_ has higher PDE activity compared to AsrR_D58E_ (Fig. 4D and E). Furthermore, when wild-type AsrR was treated with acetyl phosphate to increase its phosphorylation level *in vitro*, its PDE activity was significantly reduced compared with that without treatment, whereas acetyl phosphate treatment led to no significant change in the PDE activity of AsrR_D58A_ (Fig. 4F; Fig. S3). These results indicate that the dephosphorylated AsrR has a higher activity than phosphorylated AsrR to degrade c-di-GMP.

Then, to investigate whether AI-2 regulates intracellular c-di-GMP levels via the AsrK-AsrR TCS, the concentrations of cellular c-di-GMP were quantified in Δ*luxP* and its derivative mutants. The intracellular c-di-GMP levels in mutants Δ*luxP*Δ*luxS*, Δ*luxP*Δ*asrK,* and Δ*luxP*Δ*asrR* were significantly increased compared with Δ*luxP*, while the exogenous addition of 1 µM DPD in cultures of Δ*luxP*Δ*luxS* resulted in a significant decrease in intracellular c-di-GMP concentration (Fig. 4G and H), supporting that AI-2 inhibits the phosphorylation level of AsrR and thus activates the PDE activity of AsrR. By contrast, deletion of *luxS* in the Δ*luxP*Δ*asrK* and Δ*luxP*Δ*asrR* mutants did not lead to significant changes in the intracellular level of c-di-GMP, and the addition of 1 µM DPD did not change intracellular c-di-GMP concentration in Δ*luxP*Δ*asrK*Δ*luxS* and Δ*luxP*Δ*asrR*Δ*luxS* (Fig. 4G and H), further indicating that AI-2 decreases intracellular c-di-GMP levels via AsrK-AsrR. In addition, Δ*luxP*Δ*asrR* showed increased biofilm formation but reduced swimming motility compared to Δ*luxP*, whereas deletion of *luxS* or *asrK* in Δ*luxP*Δ*asrR* did not lead to significant changes in these phenotypes (Fig. 4I and J). These results, together with our above data (Fig. 2 and 3), suggest that AI-2 modulates intracellular c-di-GMP levels and thus regulates biofilm formation and swimming motility in *V. furnissii* via the AsrK-AsrR TCS.

### Determination of the AI-2 regulon via AsrK-AsrR system by transcriptome analysis

**Fig 5:**
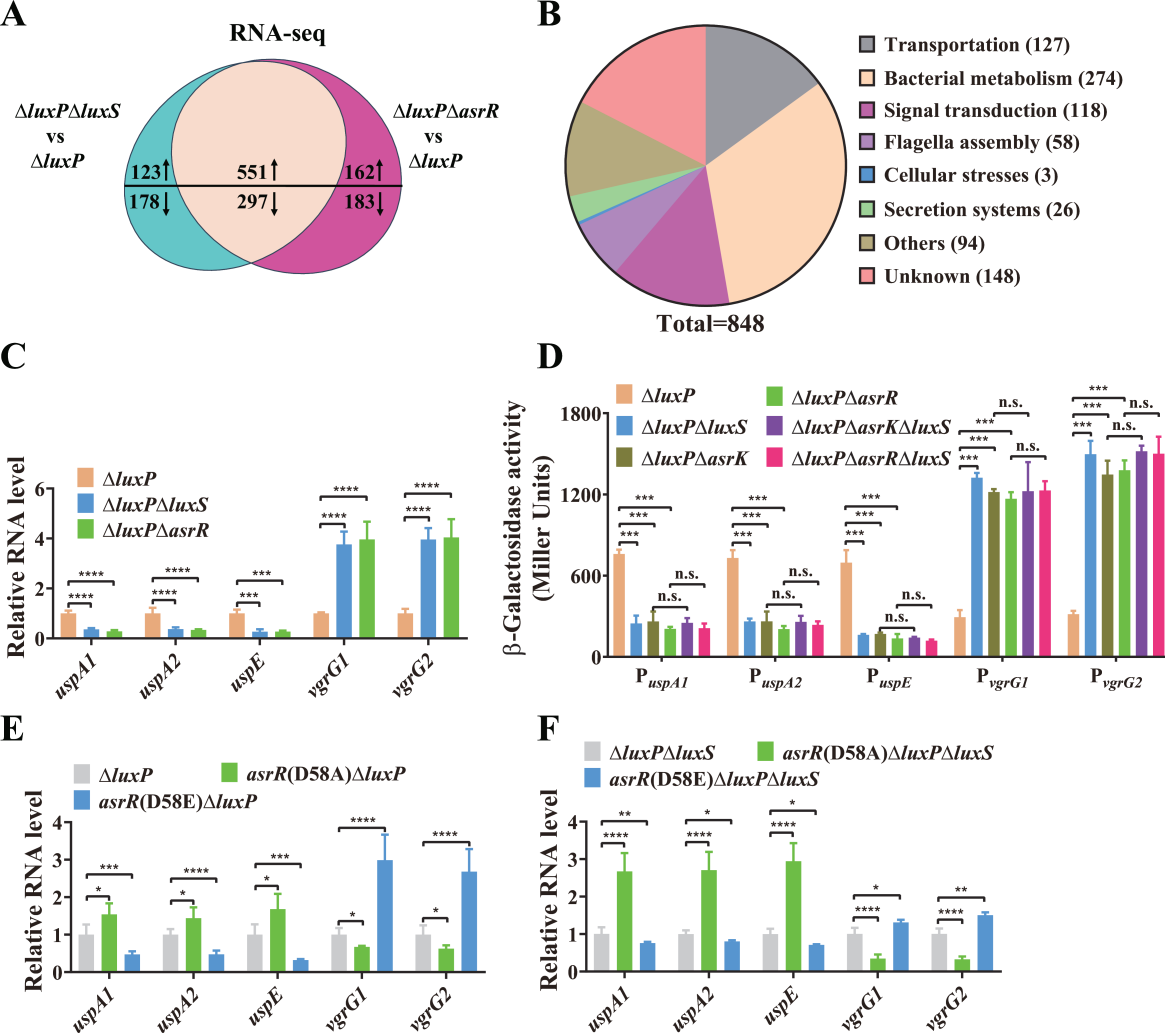
The *luxS* gene regulates the expression of numerous genes involved in diverse functions via the AsrK-AsrR TCS. (**A**) Venn diagram shows the number of unique and shared genes upregulated and downregulated (|log_2_ fold change| > 1, Benjamini–Hochberg adjusted *P*  <  0.05) in Δ*luxP*Δ*luxS* or Δ*luxP*Δ*asrR* compared to Δ*luxP* based on RNA-Seq (*n* = 3 biological replicates). *P*-values were calculated using a two-sided Wald test in the DESeq2 R package and then adjusted using the Benjamini–Hochberg correction method. (**B**) Functional classification of downstream genes regulated by both *luxS* and *asrR*. (**C**) qRT-PCR analysis of the expression of *uspA1*, *uspA2*, *uspE*, *vgrG1,* and *vgrG2* in the Δ*luxP* mutant and its derivative strains. (**D**) The promoter activities of *uspA1*, *uspA2*, *uspE*, *vgrG1,* and *vgrG2* in Δ*luxP* and its derivative mutants were measured by β-galactosidase activity assays. (**E**) qRT-PCR analysis of the expression of the three Usp genes and the two T6SS genes in the Δ*luxP* mutant and its derivative point mutants *asrR*(D58A)Δ*luxP* and *asrR*(D58E)Δ*luxP*. (**F**) qRT-PCR analysis of the expression of the three Usp genes and the two T6SS genes in the Δ*luxP*Δ*luxS* mutant and its derivative point mutants *asrR*(D58A)Δ*luxP*Δ*luxS* and *asrR*(D58E)Δ*luxP*Δ*luxS*. In panels C, E, and F, expression levels were normalized to 16S rRNA and presented as values relative to that of Δ*luxP* (**C and E**) or that of Δ*luxP*Δ*luxS* (**F**). In panels C–F, statistical analyses were carried out by results from three independent experiments, each experiment having three technical replicates. Data are mean ± s.d., and Student’s *t*-test was used for these analyses. n.s., not significant; *P < 0.05; **P < 0.01; ***P < 0.001; ****P < 0.0001.

c-di-GMP is a bacterial second messenger that transduces extracellular signals into intracellular responses and thus coordinates a plethora of important biological processes (17, 19, 20, 30, 32, 33). To investigate AI-2-regulated genes via the AsrK-AsrR TCS, we used high-throughput RNA sequencing (RNA-seq) to identify the differentially expressed genes between Δ*luxP* and Δ*luxP*Δ*luxS*, Δ*luxP*Δ*asrR* mutants. RNA-seq results showed that 475 genes were significantly downregulated, while 674 genes were significantly upregulated (absolute log_2_ fold change > 1, Benjamini–Hochberg adjusted *P* < 0.05) in Δ*luxP*Δ*luxS* compared with Δ*luxP* (Data S1). Moreover, 713 and 480 genes were significantly upregulated and downregulated, respectively, in Δ*luxP*Δ*asrR* compared with Δ*luxP* (Data S1). While deletion of *luxS* or *asrR* in Δ*luxP* led to elevated c-di-GMP levels (Fig. 4G), Δ*luxP*Δ*luxS* and Δ*luxP*Δ*asrR* shared 551 significantly upregulated genes and 297 significantly downregulated genes when compared with Δ*luxP* (Fig. 5A; Data S1). These shared downstream genes are involved in diverse functions, including transportation, bacterial metabolism, signal transduction, flagella assembly, cellular stresses, and protein secretion (Fig. 5B).

To validate the accuracy of RNA-seq results, the expression of several genes involved in stress tolerance and protein secretion (Fig. S4) was measured by quantitative real-time PCR (qRT-PCR) analysis. Consistently, the mRNA levels of the universal stress protein (Usp) genes *uspA1*, *uspA2,* and *uspE* were significantly lower, while those of T6SS genes *vgrG1* and *vgrG2* were significantly higher in Δ*luxP*Δ*luxS* and Δ*luxP*Δ*asrR* compared with Δ*luxP* (Fig. 5C). Promoter-reporter assays showed that the promoter activities of *uspA1, uspA2,* and *uspE* was significantly decreased, while those of *vgrG1* and *vgrG2* was significantly increased in Δ*luxP*Δ*luxS* compared with Δ*luxP* (Fig. 5D). Similar results were also observed in Δ*luxP*Δ*asrK* and Δ*luxP*Δ*asrR* mutants compared to Δ*luxP*, and the deletion of *luxS* in Δ*luxP*Δ*asrK* and Δ*luxP*Δ*asrR* mutants did not lead to significant changes in the promoter activities of *uspA1*, *uspA2*, *uspE, vgrG1,* and *vgrG2* (Fig. 5D). These results further prove the reliability of the RNA-seq data. Moreover, the addition of 1 µM DPD significantly increased the promoter activities of *uspA1*, *uspA2,* and *uspE,* but strongly repressed those of *vgrG1* and *vgrG2* in Δ*luxP*Δ*luxS*, whereas this effect was completely abolished in Δ*luxP*Δ*asrK*Δ*luxS* and Δ*luxP*Δ*asrR*Δ*luxS* (Fig. S5), providing further evidence that regulation of these genes is mediated by the AI-2 signal via the AsrK-AsrR TCS. Furthermore, the D58A point mutation of *asrR* in Δ*luxP* slightly upregulated the expression of *uspA1*, *uspA2,* and *uspE*, and slightly downregulated the expression of *vgrG1* and *vgrG2*, while the D58E point mutation of *asrR* resulted in remarkably decreased expression of the Usp genes and remarkably increased expression of the T6SS genes (Fig. 5E), suggesting that the majority of AsrR remains in a non-phosphorylated state in Δ*luxP*. By contrast, the D58A mutation of *asrR* in Δ*luxP*Δ*luxS* led to remarkably upregulated expression of the Usp genes and remarkably downregulated expression of the T6SS genes, whereas the expression of these genes was slightly changed by the D58E mutation of *asrR* in Δ*luxP*Δ*luxS* (Fig. 5F)*,* suggesting that the majority of AsrR should be phosphorylated in Δ*luxP*Δ*luxS.* These results are consistent with our *in vitro* data that AI-2 perception by AsrK results in reduced phosphorylation and thus increased PDE activity of AsrR. Taken together, these data indicate that AI-2 regulates the expression of genes involved in diverse functions by modulation of c-di-GMP via the AsrK-AsrR TCS.

### AI-2 enhances the defense of *V. furnissii* against oxidative stress and DNA damage via AsrK-AsrR

**Fig 6:**
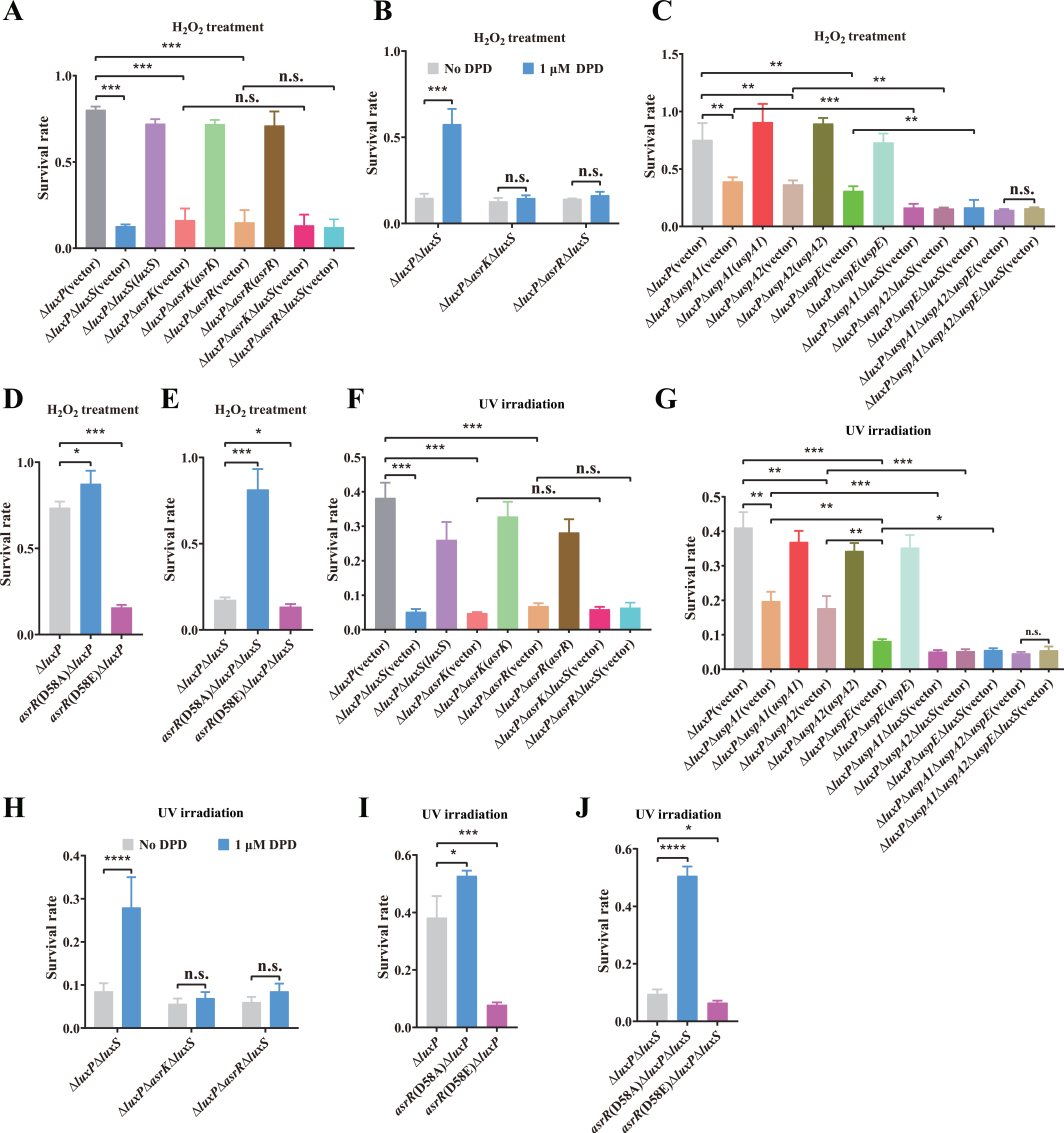
AI-2 promotes the resistance of *V. furnissii* to oxidative stress and DNA damage via the AsrK-AsrR TCS. (**A**) Survival rates of Δ*luxP*, its derivative mutants lacking *luxS*, *asrK,* or *asrR* and complemented strains after exposure to the oxidative agents H_2_O_2_. (**B**) The addition of 1 µM DPD significantly increases the H_2_O_2_ resistance of Δ*luxP*Δ*luxS* but leads to no significant changes in the H_2_O_2_ resistance of Δ*luxP*Δ*asrK*Δ*luxS* and Δ*luxP*Δ*asrR*Δ*luxS*. (**C**) The *uspA1*, *uspA2,* and *uspE* genes are all involved in cellular resistance to oxidative stress and deletion of *luxS* in Δ*luxP*Δ*uspA1*Δ*uspA2*Δ*uspE* leads to no significant changes in the H_2_O_2_ resistance. (**D**) H_2_O_2_ resistance of Δ*luxP* and its derivative point mutants *asrR*(D58A)Δ*luxP* and *asrR*(D58E)Δ*luxP*. (**E**) H_2_O_2_ resistance of Δ*luxP*Δ*luxS* and its derivative point mutants *asrR*(D58A)Δ*luxP*Δ*luxS* and *asrR*(D58E)Δ*luxP*Δ*luxS*. (**F and G**) Survival rates of the *V. furnissii* strains after exposure to UV irradiation. (**H**) The addition of 1 µM DPD significantly increases the UV resistance of Δ*luxP*Δ*luxS* but leads to no significant changes in the UV resistance of Δ*luxP*Δ*asrK*Δ*luxS* and Δ*luxP*Δ*asrR*Δ*luxS*. (**I**) UV resistance of Δ*luxP* and its derivative point mutants *asrR*(D58A)Δ*luxP* and *asrR*(D58E)Δ*luxP*. (**J**) UV resistance of Δ*luxP*Δ*luxS* and its derivative point mutants *asrR*(D58A)Δ*luxP*Δ*luxS* and *asrR*(D58E)Δ*luxP*Δ*luxS*. In panels A–J, statistical analyses were carried out by results from three independent experiments, each experiment having three technical replicates. Data are mean ± s.d., and Student’s *t*-test was used for these analyses. n.s., not significant; *P < 0.05; **P < 0.01; ***P < 0.001; ****P < 0.0001.

The Usp family of proteins UspA and UspE are involved in cellular oxidative stress response and DNA damage resistance in *E. coli* (34, 35). Based on the above observations, we speculated that AI-2 may enhance the defense of *V. furnissii* against oxidative stress and DNA damage via the AsrK-AsrR TCS. Indeed, when treated with 100 µM H_2_O_2_, the survival rates of the mutants Δ*luxP*Δ*luxS*, Δ*luxP*Δ*asrK* and Δ*luxP*Δ*asrR* were significantly decreased compared with Δ*luxP*, and complementation restored the resistance of these mutants to H_2_O_2_ (Fig. 6A). By contrast, deletion of *luxS* in Δ*luxP*Δ*asrK* and Δ*luxP*Δ*asrR* mutants did not lead to significant changes in cellular survival rates (Fig. 6A). Furthermore, the addition of 1 µM DPD significantly increased the resistance of Δ*luxP*Δ*luxS* against H_2_O_2_, but did not lead to significant changes in the H_2_O_2_ resistance of Δ*luxP*Δ*asrK*Δ*luxS* and Δ*luxP*Δ*asrR*Δ*luxS* (Fig. 6B). Moreover, the H_2_O_2_ resistance of Δ*luxP*Δ*uspA1,* Δ*luxP*Δ*uspA2* and Δ*luxP*Δ*uspE* mutants was significantly lower than that of Δ*luxP*, and the tolerance of cells to H_2_O_2_ was restored by complementation with a plasmid derived copy of *uspA1, uspA2,* or *uspE* (Fig. 6C). Deletion of the *luxS* gene in the Δ*luxP*Δ*uspA1,* Δ*luxP*Δ*uspA2,* and Δ*luxP*Δ*uspE* mutants also significantly decreased their H_2_O_2_ resistance (Fig. 6C). However, when all of the three genes *uspA1*, *uspA2,* and *uspE* were absent, no significant change in H_2_O_2_ resistance was observed between Δ*luxP*Δ*uspA1*Δ*uspA2*Δ*uspE* and Δ*luxP*Δ*uspA1*Δ*uspA2*Δ*uspE*Δ*luxS* (Fig. 6C). In addition, the D58A and D58E point mutations of *asrR* slightly increased and remarkably decreased, respectively, the H_2_O_2_ resistance of Δ*luxP* (Fig. 6D), while these two point mutations in Δ*luxP*Δ*luxS* led to a remarkable increase and slight decrease of the H_2_O_2_ resistance, respectively (Fig. 6E). These results indicate that AI-2 improves cellular resistance to oxidative stress by promoting expression of *uspA1*, *uspA2,* and *uspE* via AsrK-AsrR.

Meanwhile, we found that the resistance to UV irradiation in mutants Δ*luxP*Δ*luxS*, Δ*luxP*Δ*asrK*, Δ*luxP*Δ*asrR*, Δ*luxP*Δ*uspA1*, Δ*luxP*Δ*uspA2,* and Δ*luxP*Δ*uspE* was significantly decreased compared with Δ*luxP*, and complementation restored their resistance to UV irradiation (Fig. 6F and G). Interestingly, the mutants Δ*luxP*Δ*uspA1* and Δ*luxP*Δ*uspA2* were less sensitive to ultraviolet light than the Δ*luxP*Δ*uspE* mutant (Fig. 6G), suggesting that UspE plays a more important role than UspA1 and UspA2 in providing the cell with resistance against UV irradiation. Deletion of *luxS* in Δ*luxP*Δ*uspA1,* Δ*luxP*Δ*uspA2* and Δ*luxP*Δ*uspE* significantly decreased their UV resistance (Fig. 6G), and Δ*luxP*Δ*uspA1*Δ*uspA2*Δ*uspE* and Δ*luxP*Δ*uspA1*Δ*uspA2*Δ*uspE*Δ*luxS* showed similar sensitive to UV exposure (Fig. 6G). Moreover, the addition of 1 µM DPD significantly enhanced the UV resistance of Δ*luxP*Δ*luxS* but led to no significant changes in that of Δ*luxP*Δ*asrK*Δ*luxS* and Δ*luxP*Δ*asrR*Δ*luxS* (Fig. 6H). Furthermore, the D58E point mutation of *asrR* remarkably decreased the UV resistance of Δ*luxP*, whereas the D58A point mutation of *asrR* remarkably increased the UV resistance of Δ*luxP*Δ*luxS* (Fig. 6I and J). These data suggest that AI-2 also participates in protecting cells against DNA damage via upregulating expression of *uspA1*, *uspA2,* and *uspE*. Together, our results indicate that AI-2 enhances the defense of *V. furnissii* against oxidative stress and DNA damage by regulating expression of the Usp proteins via AsrK-AsrR.

## DISCUSSION

**Fig 7:**
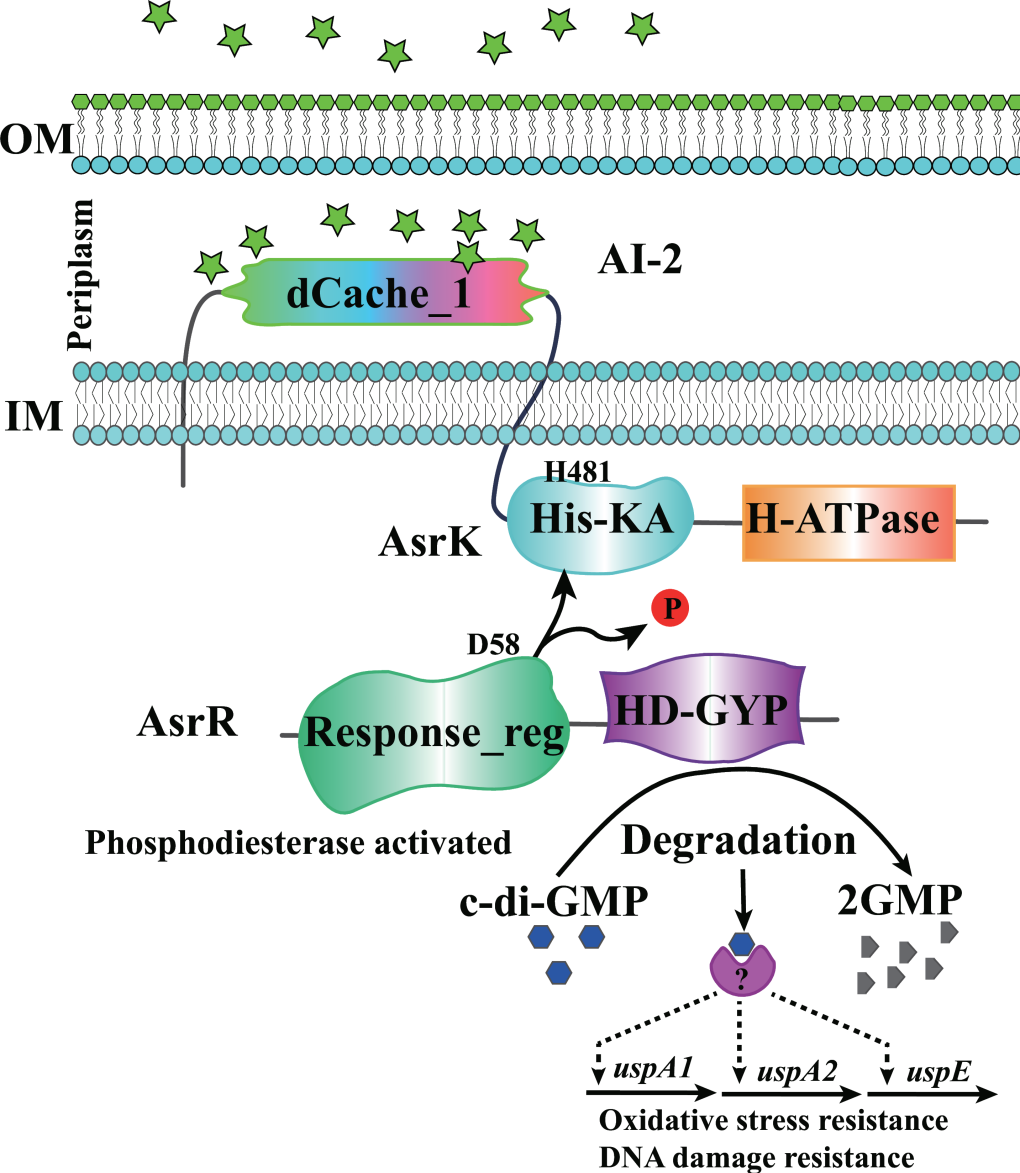
A model of perception of AI-2 by the AsrK-AsrR TCS. The QS signal AI-2 binds to the periplasmic dCache_1 domain of the HK AsrK to inhibit its autokinase activity, thus leading to decreased phosphorylation of its cognate response regulator AsrR. Dephosphorylation activates the PDE activity of AsrR in degrading c-di-GMP. When the level of c-di-GMP in the cell is reduced, the transcription of *uspA1*, *uspA2,* and *uspE* is upregulated to increase cellular resistance to oxidative stress and DNA damage.

The QS signal AI-2 is widely distributed throughout the bacterial kingdom and has been implicated in the regulation of a variety of niche-speciﬁc behaviors (17, 36). However, its two earlier identified receptors LuxP and LsrB are only found in a small number of bacterial species capable of responding to this signaling molecule (14, 15, 37–49). In particular, LuxP is the only AI-2 receptor ever discovered in *the Vibrionales* order. Here, we identify the transmembrane HK AsrK as an AI-2 receptor in *V. furnissii*. Perception of AI-2 under low boron conditions by the periplasmic dCache_1 domain of AsrK inhibits its autokinase activity and decreases the phosphorylation level of its cognate RR AsrR, which, in turn, activates the phosphodiesterase activity of the latter in degrading c-di-GMP (Fig. 7). Thus, AI-2 reduces intracellular c-di-GMP levels via the AsrK-AsrR TCS and regulates the transcription of hundreds of genes involved in diverse biological processes. Among them, three Usp genes are positively regulated by AI-2-triggered activation of AsrR and enhance the tolerance of *V. furnissii* to oxidative stress and DNA damage. Our study establishes a direct connection between AI-2 and c-di-GMP signaling in the *Vibrio* spp. and also expands our understanding of the physiological roles of AI-2 in bacteria.

Previous studies have revealed various regulatory connections between QS and c-di-GMP signaling (10, 17, 30, 50). QS signals, including AHLs, diffusible signal factors (DSFs), and AI-2, can be integrated into the c-di-GMP signaling network by regulating the expression or activity of c-di-GMP-metabolizing enzymes via direct or indirect means (10, 17, 25, 28, 30). The AHL signal produced by the Las QS system upregulates expression of the tyrosine phosphatase TpbA, and the latter dephosphorylates the DGC TpbB, thus leading to reduced c-di-GMP production in *P. aeruginosa* (28). Perception of DSF by the sensor HK RpfC leads to phosphorylation of its cognate RR RpfG, which activates its PDE activity and decreases the c-di-GMP levels in *Xanthomonas campestris* (25). The DGC YeaJ directly senses AI-2 via its periplasmic GAPES1 domain to stimulate its activity and increase intracellular c-di-GMP levels in *S*. Typhimurium (17). In *V. cholerae,* the increase in AI-2 level is sensed by LuxP and modulates a TCS phosphorelay cascade that results in dephosphorylation of the transcriptional regulator LuxO, controlling c-di-GMP levels indirectly through altering the expression of c-di-GMP-metabolizing enzymes (30). However, the Scr QS system of *Vibrio parahaemolyticus* directly controls the activity of a bifunctional c-di-GMP synthesis or degradation enzyme (51). Our current study shows that AI-2 directly regulates the activity of an HD-GYP enzyme via a TCS phosphorelay cascade and leads to a decrease in c-di-GMP levels, thus revealing a previously unrecognized direct connection between QS and c-di-GMP in members of *Vibrio*. Nevertheless, the integration of QS and c-di-GMP has only been studied in a few bacterial species, and it is expected that the list will continue to grow.

Although AI-2 is a well-conserved QS signal in both Gram-negative and Gram-positive bacteria (10), its clear role in QS has been demonstrated only in a small subset of bacteria producing or responding to this signal (49, 52), largely due to the fact that the AI-2 receptor LsrB that mediates AI-2 internalization, chemotaxis, and cell aggregation is found only in a small number of bacterial species (8, 48, 49, 52, 53) while another AI-2 receptor LuxP is present only in *Vibrio* spp. (11). While AI-2 has been shown to regulate bacterial functions including bioluminescence, biofilm formation, motility, virulence factor production, competence, and antibiotic resistance (6, 8, 10, 12, 13, 17, 54–57), the underlying mechanisms are still unknown in a large number of bacteria lacking LuxP- or LsrB-like AI-2 receptors. In two recent studies, we identified dCache_1 domain- and GAPES1 domain-containing signal transduction proteins as AI-2 receptors (10, 17), and thus greatly expanded the possibility to explore unknown functions and molecular mechanisms involved in AI-2-mediated signaling. In the present study, we further identify a previously unknown dCache_1 domain-containing AI-2 receptor in *V. furnissii* and first report the regulatory function of AI-2 in defense against oxidative stress and DNA damage. Given the global influence of c-di-GMP signaling on bacterial physiology, as well as a large number of genes regulated by the AI-2-AsrKR regulatory cascade (Fig. 5), the physiological roles of AI-2 in *V. furnissii* remain to be explored.

In summary, our work identifies a second type of AI-2 receptor in a member of *Vibrio* and reveals how the signaling pathway mediated by this receptor senses and responds to the AI-2 signal to regulate important functions such as the defense against oxidative stress and DNA damage. Our study reveals a previously unrecognized mechanism by which AI-2 directly modulates c-di-GMP-metabolizing enzyme activity in bacteria. These findings highlight not only the complexity of AI-2 signaling networks but also the functional diversity of this signal. Nevertheless, in contrast to the widespread distribution of this signal in bacteria, our understanding of the role of AI-2 is obviously insufficient. Further research is needed to better understand the multiple regulatory effects and regulatory mechanisms of AI-2 in a wider range of bacteria.

## MATERIALS AND METHODS

### Bacterial strains and growth conditions

The bacterial strains and plasmids used in this study are listed in Table S1. The *E. coli* strains were grown at 37°C in either LB or TSB medium. *V. harveyi* MM32 (*luxN^−^*, *luxS*^−^) was grown in AB medium (58) at 30°C. The *V. furnissii* strains were grown at 30°C in either LB or LMB (LB supplemented with 2% NaCl) unless specified otherwise. Antibiotics were used at the following concentrations for *V. furnissii*: chloramphenicol (20 µg/mL) and gentamicin (10 µg/mL). Antibiotics were used at the following concentrations for *E. coli*: kanamycin (50 µg/mL) and chloramphenicol (20 µg/mL).

### Plasmid construction

Primers used in this study are listed in Table S2. To construct in-frame deletion and point mutants, the pDM4 derivatives were transformed into relevant *V. furnissii* strains through *E. coli* S17-1-mediated conjugation and were screened as described by Zhang et al. (59). For complementation, the pBAD22 derivatives were transformed into relevant *V. furnissii* strains separately and induced by the addition of 10 mM arabinose. To express and purify soluble His_6_-tagged recombinant proteins, genes were cloned into pET-28a, transformed into *E. coli* BL21(DE3) host strains, and induced by the addition of 0.25 mM isopropyl-β-D-1-thiogalactopyranoside (IPTG). To construct a promoter *lacZ* transcription fusion reporter strain, gene promoter regions were individually cloned into the vector pDM4-*lacZ,* and the resulting constructs were separately transformed into *E. coli* strain S17-1λpir. The *E. coli* S17-1λpir derivatives carrying the promoter-*lacZ* fusion reporter plasmids were mated with *V. furnissii* strains, and then the transconjugants were selected on LB agar containing gentamicin and chloramphenicol (19).

### Boron removal from reagents and media

To remove boron, water, the media, and Tris buffer were filtered through a borate anion-specific resin Amberlite IRA-743 (Sigma cat# 216445) (60). In brief, 30 mL of the borate anion-specific resin is used to remove boron from 1,000 mL of solution in a 50 mL polypropylene column by going through the following steps in sequence: 150 mL of 3 M NH_4_OH, 600 mL of distilled water, 300 mL of 1 M HCl, 150 mL of distilled water, 300 mL of 0.16 M HNO_3_, 600 mL of distilled water, and 1,000 mL of the solution. All liquid was allowed to filter through the resin at an effluent rate of ~2 drops s^−1^. For all experiments involving borate-depleted reagents or media, only plastic supplies were used. In all experiments that did not involve boron depletion, cultures were grown in glassware including test tubes and triangular bottles.

### β-Galactosidase assays

The β-galactosidase assays were modified from a previously reported study (19, 59). The absorbance of bacterial culture at 600 nm was measured. A total of 150 µL of bacterial culture was added to 630 µL of Z-buffer (40 mM NaH_2_PO_4_; 10 mM KCl; 60 mM Na_2_HPO_4_; 1 mM MgSO_4_; and 5.4 µL/mL β-mercaptoethanol) and mixed thoroughly. Thirty microliters of chloroform and 15 µL of 0.1% sodium dodecyl sulfate (SDS) were added to the mixture, vigorously mixed again for 30 s, and then incubated at 30°C for 1 h. Preincubated substrate solution (150 µL; 60 mM Na_2_HPO_4_; 40 mM NaH_2_PO_4_; 4 mg/mL 2-nitrophenyl-β-D-galactopyranoside; and 2.7 µL/mL β-mercaptoethanol) was added to the mixture for reaction. The reaction was stopped by adding 375 µL of 1 M Na_2_CO_3_. The suspension was centrifuged at 10,000 × *g* for 3 min, and the absorbance of the supernatant was read at 420 and 550 nm using a microplate reader. The β-galactosidase activity was calculated and expressed as Miller units (61). For AI-2 signal supplementation, the DPD solution was added at the time of inoculation.

### Biofilm formation assays

The biofilm formation assays were performed as described previously with some modifications (17, 21). Briefly, *V. furnissii* strains were grown overnight in LMB liquid medium at 30°C with shaking at 200 rpm, and the culture was diluted with fresh LMB to an absorbance of 0.05 at 600 nm. 100 µL of diluted cell culture was added to each well of the sterile 96-well polystyrene microplate and statically cultured at 30°C for 24 h. Then, culture supernatants were removed, and the wells were washed twice with sterilized PBS. Two hundred microliters of 0.1% (wt/vol) crystal violet staining solution was added to each well and incubated for 10 min, after which the wells were washed twice with sterilized PBS to remove unbound crystal violet dye. Ethanol (200 µL) was added to each well to dissolve bacteria-bound dye, and the absorbance was read at 590 nm. For AI-2 signal supplementation, the DPD solution was added at the time of inoculation. To test the role of boron, *V. furnissii* strains were grown in a borate-depleted LMB medium with or without 5 mM boric acid in the presence or absence of 1 µM DPD diluted in borate-depleted water.

### Swimming motility assays

The swimming motility assays were performed according to previously described methods (17, 21). Briefly, *V. furnissii* strains were cultured overnight in LMB liquid medium at 30°C and the culture was diluted with fresh LMB to an absorbance of 2 at 600 nm. Two microliters of the diluted bacterial culture was spotted onto 0.3% tryptone agar (1% tryptone; 0.5% NaCl; and 0.3% Bacto agar [Difco]) in plastic plates. Motility halos were measured after the plates were incubated at 30°C for 12 h. For AI-2 signal supplementation, the DPD solution was added to the swimming motility medium before the agar solidified (40°C–45°C). To test the role of boron, *V. furnissii* strains were grown in borate-depleted swimming motility medium (Bacto agar was washed three times with borate-depleted water before being mixed with the other borate-depleted medium components) with or without 5 mM boric acid in the presence or absence of 1 µM DPD diluted in borate-depleted water.

### Overexpression and purification of recombinant proteins

The overexpression and purification of recombinant proteins were performed using previously described methods (19). *E. coli* BL21(DE3) strains carrying the pET-28a derivatives were cultured in LB liquid medium at 37°C and 200 rpm until an OD_600_ of 0.8, and then 0.25 mM IPTG was added to induce protein expression at 16°C for 12 h. Bacterial cells were harvested and lysed by sonication, and then recombinant proteins were purified with Ni-nitrilotriacetic acid (Ni^2+^-NTA) His-bind resin (Novagen, Madison, WI) according to the manufacturer’s instructions. Subsequently, the purified proteins were dialyzed using Tris buffer (50  mM Tris; 150  mM NaCl and 10% glycerol, pH 7.5) at 4°C. The purity of proteins was evaluated by SDS-PAGE and the protein concentrations were determined using the Bradford assay with bovine serum albumin as standard.

### Molecular docking analysis

The three-dimensional structure of the dCache_1 domain of AsrK was predicted by AlphaFold2 (https://colab.research.google.com/github/sokrypton/ColabFold/blob/main/AlphaFold2.ipynb#scrollTo=kOblAo-xetgx) (62). The active AI-2 molecule form R-THMF was extracted from the crystal structure of the R-THMF-LsrB (PDB ID: 1TJY) complex (12), and its flexible torsions were assigned using Autodock4 (https://autodock.scripps.edu/download-autodock4) (63). Docking simulation between R-THMF and the pocket in the membrane-distal module of AsrK-LBD was done with AutoDock Vina 1.1.2 (https://vina.scripps.edu) (64), and the best binding mode was selected based on the lowest docking energy. The three-dimensional figure of the R-THMF-AsrK-LBD was displayed using PyMOL (http://www.pymol.org).

### *In vitro* AI-2 binding assays

The *in vitro* AI-2 binding assays were performed as described previously (10, 17). The pET-28a derivatives carrying the DNA fragment encoding the dCache_1 domains were transformed into *E. coli* strain BL21(DE3) or its mutant lacking *luxS* (10). The resulting strains were cultured in LB liquid medium at 37°C until an OD_600_ of 0.8, and then induced with 0.25 mM IPTG at 16°C for 8 h. After being purified by Ni^2+^-NTA His-bind resin according to the manufacturer’s instructions, the proteins in elution buffer were swapped into a solution containing 50 mM NaH_2_PO_4_ (pH 8.0), 300 mM NaCl, and 1 mM dithiothreitol (DTT) using Sephadex G-25. Protein purity was assessed by SDS-PAGE, and the purified proteins were concentrated to ~10 mg mL^−1^, followed by denaturation via heating at 70°C for 10 min to release any bound ligands. The denatured proteins were precipitated, and the supernatants were collected to determine the presence or absence of AI-2 using the *V. harveyi* MM32 luminescence assay (10). Bioluminescence in *V. harveyi* MM32 was induced by ligands released from purified proteins expressed in *E. coli* BL21(DE3) or its Δ*luxS* mutant, and AI-2 activity is reported as fold induction over background obtained in the buffer control alone.

### ITC analysis

ITC experiments were performed at 20°C using the Nano-ITC standard volumetric isothermal calorimeter (TA Instruments, New Castle, DE). Purified AsrK-LBD, AsrK-LBD_Y213A_, AsrK-LBD_D215A_, and AsrK-LBD_D242A_ were dialyzed with Tris buffer (25 mM Tris-HCl and 150 mM NaCl, pH 7.5) which was stored in a plastic bottle and then diluted to 10 µM, and the DPD solution (Omm Scientific) was diluted in the same buffer to 150 µM. After being degassed, 1 mL of the protein and 250 µL of DPD were added to the sample cell and syringe, respectively. There were 25 injections per experiment and three independent experiments on each protein sample were performed. For control experiments, DPD was titrated into the buffer in the sample cell. After subtracting the heat of dilution, the microcalorimetric data were fit to an independent binding model by the NanoAnalyze software (10). To test the effect of boron on the interactions of AI-2 with vfu_B00258-LBD, the DPD solution (3.42 mM) was diluted with borate-depleted Tris buffer to a concentration of 30 µM, and the vfu_B00258-LBD protein was dialyzed against and diluted in the borate-depleted Tris buffer to a concentration of 2 µM. The borate-depleted DPD (30 µM) was titrated with the borate-depleted vfu_B00258-LBD (2 µM), while the borate-depleted DPD (30 µM) supplemented with 150 µM boric acid was titrated with the borate-depleted vfu_B00258-LBD (2 µM) supplemented with 150 µM boric acid. The heats of dilution for the borate-depleted DPD titrated into the borate-depleted buffer were subtracted from the raw titration data of borate-depleted vfu_B00258-LBD in response to borate-depleted DPD before analysis, while the heats of dilution for the borate-depleted DPD with added boric acid titrated into the borate-depleted buffer with added boric acid were subtracted from the raw titration data of borate-depleted vfu_B00258-LBD with added boric acid in response to borate-depleted DPD with added boric acid before analysis.

### Preparation of the transmembrane proteins AsrK, AsrK_Y213A_ and AsrK_D215A_

The preparation of full-length AsrK, AsrK_Y213A_, and AsrK_D215A_ was performed as described previously (17, 27). The *asrK* gene and its point mutant *asrK*_Y213A_ and *asrK*_D215A_ were cloned with an N-terminal His_6_ tag into pET-28a, and the expression constructs were transformed into the ∆*luxS* mutant of *E. coli* BL21(DE3). After IPTG induction, bacterial cells were harvested and lysed, followed by collection of the supernatants via centrifugation at 10,000 × *g* for 10 min at 4°C. The membrane fractions in the supernatants were precipitated by ultracentrifugation at 200,000 × *g* for 1 h at 4°C and then resuspended in a high-salt buffer (20 mM Na_3_PO_4_, pH 7.0; 2 M KCl; 10% glycerol; 5 mM EDTA; 5 mM DTT; and 1 mM phenylmethanesulfonyl fluoride), followed by three rounds (1 h per round) of ultracentrifugation at 350,000 × *g* and 4°C. The membrane fractions containing full-length AsrK and AsrK_D215A_ in the high-salt buffer were further purified using Ni^2+^-NTA affinity chromatography and subjected to SDS-PAGE to evaluate the protein purity. The total protein concentration was measured using the Bradford method.

### *In vitro* phosphorylation assays

Non-radioactive *in vitro* autokinase and phosphotransfer assays were conducted as described previously (65–69) with minor modifications. Briefly, to detect autophosphorylation of AsrK, 0.25 µM purified His_6_-tagged AsrK, AsrK_Y213A_ or AsrK_D215A_ was incubated in EP tubes with 250 µM ATP-γ-S (ab138911, Abcam) in 20 µL of kinase reaction buffer (50 mM Tris, pH 7.4, and 10 mM MgCl_2_) which was stored in a glass bottle for 30 min at 30°C. When needed, DPD (0, 2, and 10 µM) was added simultaneously with ATP-γ-S. The thiophospholyation site on AsrK was then alkylated by adding 2.5 mM p-nitrobenzyl mesylate (ab138910, Abcam) for 1 h at 30°C, and the reaction was stopped with 5× SDS loading buffer. To test a possible role of boron in the effect of AI-2 on the autokinase activity of AsrK, the purified AsrK protein was dialyzed against and diluted in borate-depleted Tris buffer, and the DPD solution was diluted with the borate-depleted Tris buffer. The autokinase assay was performed in a borate-depleted reaction system with or without added 50 µM boric acid in the presence of 0, 2, and 10 µM borate-depleted DPD. To detect phosphoryl transfer between AsrK and AsrR, 0.25 µM AsrK and AI-2 (0 and 10 µM) were incubated in the 20 µL of reaction buffer that contains 250 µM ATP-γ-S for 5 min at 30°C, and then 20 µM AsrR was added to the reaction mixture, which was incubated for another 5 min at 30°C. After the phosphotransfer reaction, p-nitrobenzyl mesylate was added to alkylate the thiophospholyation site on the substrates and then the reaction was stopped with 5× SDS loading buffer. The thiophosphate ester-modified proteins in autokinase and phosphotransfer assays were separated by SDS-PAGE and transferred to the PVDF membrane using a Bio-Rad wet transfer system. The membrane was blocked with QuickBlockBlocking Buffer for 1 h at room temperature, and incubated overnight at 4°C with rabbit anti-thiophosphate ester antibody (ab92570, Abcam). The membrane was then washed six times with TBST before being incubated with a goat anti-rabbit HRP-conjugated secondary antibody (Cat No. SA00001-2, Proteintech) for 1 h at room temperature. The membrane was washed six times with TBST, and proteins were detected using an ECL detection reagent. The SDS-PAGE gel was also stained with Coomassie brilliant blue (CBB) to ascertain the migration of the proteins.

### Extraction and quantification of cellular c-di-GMP levels

The cellular c-di-GMP quantification by LC-MS/MS was performed as described previously (17). *V. furnissii* strains were grown in LMB medium at 30°C and 200 rpm until an OD_600_ of 1.5. For stimulation with AI-2, 1 µM DPD was added to the shaking culture at OD_600_ of 1.5 and incubated at 30°C for 30 min. The bacterial cells were harvested, washed twice with sterile water, and resuspended in 500 µL of extraction solution (methanol:acetonitrile:water = 40:40:20, volume ratio). After cooling on ice for 15 min, the samples were boiled for 10 min at 95°C, followed by cooling again on ice for another 15 min. Then, the samples were centrifuged at 4°C and 12,000 rpm for 10 min, and the supernatants were collected. The remaining cell precipitates were subjected to two more extraction steps using 500 µL of the extraction solution with cooling on ice but without heating. The supernatants from the three rounds of extraction were pooled, lyophilized, and resolubilized in distilled water. Then, c-di-GMP concentrations were determined by LC-MS/MS (AB SCIEX QTRAP 6500+ LC MS/MS System). Different concentrations of c-di-GMP (Sigma) were used to establish the standard curve, and the intracellular levels of c-di-GMP were normalized to the number of bacterial cells in each sample.

### *In vitro* PDE activity assays

*In vitro* PDE activity analysis was performed using previously described methods (10, 17). 50 µM AsrR or its variants were added to 200 µL of reaction buffer containing 50 mM Tris HCl (pH 7.5) and 5 mM MgCl_2_. The reaction was initiated by adding 50 µM c-di-GMP and immediately incubating at 30°C. To evaluate the effect of phosphorylation of AsrR on its PDE activity, the purified wild-type AsrR and AsrR_D58A_ were pretreated by 25 mM acetyl phosphate for 30 min at 30°C before their reaction with c-di-GMP. After incubation for 30 min, reactions were terminated by heating the samples at 100°C for 10 min. Denatured protein was removed by centrifugation, and the supernatant was filtered through a 0.22-µm filter membrane. Samples were subjected to HPLC analysis with a C18 reversed-phase column and a UV detector. The reaction products were eluted isocratically at a flow rate of 1 mL min^−1^ with a mobile phase consisting of 98% A (150 mM Na_2_HPO_4_, pH 5.2) and 2% B (acetonitrile), and the detection wavelength was 254 nm. GMP (Sigma) and c-di GMP (Sigma) were run as standards, and the levels of GMP produced in the reaction were determined based on the standard curve established using known concentrations of GMP.

### RNA extraction and qRT-PCR analysis

RNA extraction and qRT-PCR analysis were performed using previously described methods (19). *V. furnissii* strains were cultured in LMB medium until reaching an OD_600_ of 2.0. Total RNA was extracted using MolPure Bacterial RNA Kit (Yeasen Biotechnology, Shanghai, China). Reverse transcription of RNA was performed using TransScript II One-Step gDNA Removal and cDNA Synthesis SuperMix (TransGen Biotech, Beijing, China), and then qRT-PCR was performed using the TransStart Green qPCR SuperMix kit (TransGen Biotech, Beijing, China) in a LightCycle 96 thermal cycler (Roche). 16S rRNA gene was used as an internal control to normalize gene expression levels.

### High-throughput RNA-seq analysis

*V. furnissii* strains were cultured in LMB medium until reaching an OD_600_ of 2.0 before harvest. Total RNA was extracted using Trizol reagent (Invitrogen Life Technologies), and the RNA concentration, purity, and integrity were determined using a NanoDrop spectrophotometer (Thermo Scientific) and the biological analyzer 2100 system (Agilent). Ribosomal RNA was removed using the Zymo Seq RiboFree Total RNA Library Kit (Zymo Research) according to the manufacturer’s instructions. cDNA libraries were synthesized using a SuperScript III reagent kit (Invitrogen Life Technologies) and library fragments were purified using the AMPure XP system (Beckman Coulter, Beverly, CA, USA). Then, the libraries were sequenced on the NovaSeq 6000 platform (Illumina) by Shanghai Personal Biotechnology Co., Ltd. Sequence reads were mapped to the *V. furnissii* NCTC 11218 reference genome (CP002377 and CP002378) using Bowtie version 2.5.1. Read counts for each gene were counted by HTSeq v0.9.1, and then FPKM (fragments per kilobase of transcript per million mapped reads) was calculated to estimate gene expression levels. Differential gene expression analysis was conducted using DESeq2 R software v1.38.3. *P* values were adjusted by the Benjamini–Hochberg approach to control the false discovery rate (FDR). The adjusted *P*-value < 0.05 and |log_2_ fold change| > 1 were considered statistically significant.

### Determination of bacterial survival rates under H_2_O_2_ stress conditions

The H_2_O_2_ oxidative stress assays were performed as described previously with some modifications (70). *V. furnissii* strains grown overnight in LMB liquid medium at 30°C were subcultured at a 1:100 dilution in fresh LMB medium until an OD_600_ of 0.5. The cultures were diluted to OD_600_ of 0.1 by fresh LMB medium, added with 100 µM H_2_O_2_ or a buffer control, and then incubated at 30°C and 100 rpm for 1 h. Then, the unstressed cultures and the stress-exposed cultures were serially diluted, spread on LB agar plates, and incubated at 30°C for 3 days prior to CFU enumeration. The survival rate was calculated as the ratio of the number of CFUs of the stressed cells to the number of CFUs of the unstressed cells. To test the effect of the AI-2 signal on the H_2_O_2_ resistance of *V. furnissii* strains, the medium was supplemented with 1 µM DPD at the time of inoculation.

### UV stress assays

The UV stress assays were performed as described previously (34). The overnight cultures of *V. furnissii* strains were diluted at 1:100 with fresh LMB medium, and then grown to an OD_600_ of 2.0. The cultures were exposed to 50 mJ cm^−2^ of UV light (254 nm) at a dose rate of 0.5 mJ cm^−2^ s^−1^. Then, the irradiated cultures were 10-fold serially diluted and spread on LB agar plates to determine the number of viable cells. The survival rate was calculated by the ratio between irradiated cells and unirradiated cells. To test the effect of AI-2 on the UV resistance of *V. furnissii* strains, the medium was supplemented with 1 µM DPD at the time of inoculation.

### Statistical analysis

All of the experiments were performed at least three times on different occasions. GraphPad Prism 7 software (GraphPad Software Inc.; San Diego, CA, USA) and Adobe Illustrator 2020 (CS6; Adobe, Mountain View, CA, USA) are used to create all graphics. The data from RNA-seq were analyzed by the Wald test in the DESeq2 R package, and the resulting *P*-values were adjusted using the Benjamini-Hochberg FDR. All other experiments were analyzed using a two-sided, unpaired Student’s *t*-test, with the data represented as mean ± s.d. A *P*-value less than 0.05 was considered statistically significant.

## Data Availability

RNA-seq data from this study have been submitted to the NCBI Sequence Read Archive (SRA) under the accession number PRJNA1116534.

## SUPPLEMENTAL MATERIAL

Data S1 mbio.02922-24-s0001.xlsx Genes upregulated and downregulated in Δ*luxP*Δ*luxS* or Δ*luxP*Δ*asrR* compared to Δ*luxP* based on RNA-Seq.

Supplemental material mbio.02922-24-s0002.pdf Fig. S1 to S5; Tables S1 and S2.

ASM does not own the copyrights to Supplemental Material that may be linked to, or accessed through, an article. The authors have granted ASM a non-exclusive, world-wide license to publish the Supplemental Material files. Please contact the corresponding author directly for reuse.
