# Supplementary material for: Autoinducer-2 enhances the defense of Vibrio furnissii against oxidative stress and DNA damage by modulation of c-di-GMP signaling via a two-component system
Source: mBio. 2025 Jan 16;16(2):e02922-24. doi: 10.1128/mbio.02922-24 (PMC11796354; doi:10.1128/mbio.02922-24)
Supplement: Supplemental material — Fig. S1 to S5; Tables S1 and S2. [file mbio.02922-24-s0002.pdf]

```
vfu_B00258-LBD   SVDNMRYINEQVNNELKLSSQRETEIIGEKLKNIASLTALYRHETERIFATPVGEIDTEK
PctA-LBD         ---NDYLQRNAIREDLESYLREMGDVTSSNIQNWLGGRLLLVEQTAQTLAR---DHSPET

vfu_B00258-LBD   ANLQLSESGVLYSLRDEGGAASYYSGVTTNKHMEKVYQLASLDPLMKQIKENNDLVAAVY
PctA-LBD         VSALLEQPALTSTFS-----FTYLG------QQDGVFTMRPDSP-MP-----------AG

vfu_B00258-LBD   FNSWDSYNRIYPWFSTLAQYPPDMNIPDYNFYYLATYPYNPQKTGVWTDVYIDPAGLGWM
PctA-LBD         YDP-----RSRPWYKDAVAAG----------------------GLTLTEPYVDAATQELI

vfu_B00258-LBD   ASSIAPVYHHGFLEGVVGLDITVSAIVESIQHLSVPWNGYAVLASNTGTMMALPPQGEQD
PctA-LBD         ITAATPVKAAGNTLGVVGGDLSLKTLVQIINSLDFSGMGYAFLVSGDGKILVHP---DKE

vfu_B00258-LBD   FGMQELTEHNYQQAITQEVFKPDQFNLHKRPDTAILSQQLSQSAQGITQMTLQGAKKLVA
PctA-LBD         QVMKTLSE--------------------------VYPQNTPKIATGFSEAELHGHTRILA

vfu_B00258-LBD   WATIP---ETKWQLLMIVDESKMFAESRLLEHKYQR
PCTA-LBD         FTPIKGLPSVTWYLALSIDKDKAYAMLSKFRVSA--
```

Fig. S1 Sequence alignment of the LBDs of AsrK-LBD and PctA-LBD

Amino acids 43-375 and 30-278 of AsrK and PctA, respectively, were subjected to multiple sequence alignment using the ClustalW tool of the NPSA suite (https://npsa-prabi.ibcp.fr/cgi-bin/npsa_automat.pl?page=/NPSA/npsa_server.html). Red, identical; green, highly similar; blue, weakly similar. The five key residues R126, W128, Y144, D146 and D173 of PctA that are important for AI-2 binding (1-3) are highlighted in yellow. The three residues Y213, D215, and D242 of AsrK-LBD predicted to be involved in AI-2 binding (Fig. 2E) and corresponding to Y144, D146 and D173 of PctA are also highlighted in yellow.

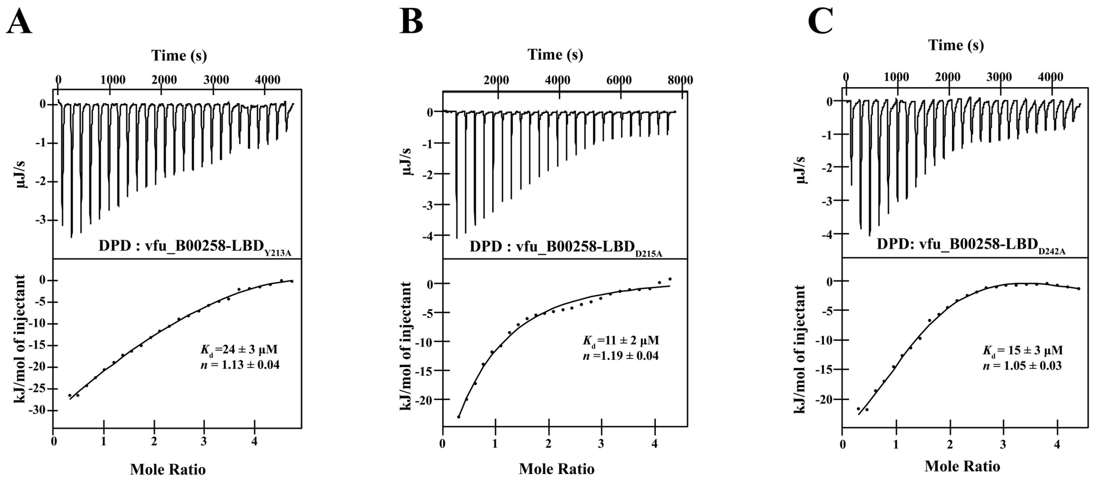

Fig. S2 AI-2 shows low affinity to vfu_B00258-LBD$_{Y213A}$, vfu_B00258-LBD$_{D215A}$ and vfu_B00258-LBD$_{D242A}$.

(A-C) The binding affinity of AI-2 to vfu_B00258-LBD$_{Y213A}$ (A), vfu_B00258-LBD$_{D215A}$ (B) and vfu_B00258-LBD$_{D242A}$ (C) evaluated using ITC analysis. ITC data and plots of injected heat for injections of 150 μM DPD into the sample cell containing 10 μM protein are shown in the upper and lower plots, respectively. Data shown are one representative of three independent experiments with similar results, and the K_d and n values are presented as mean ± s.d. of the three independent experiments.

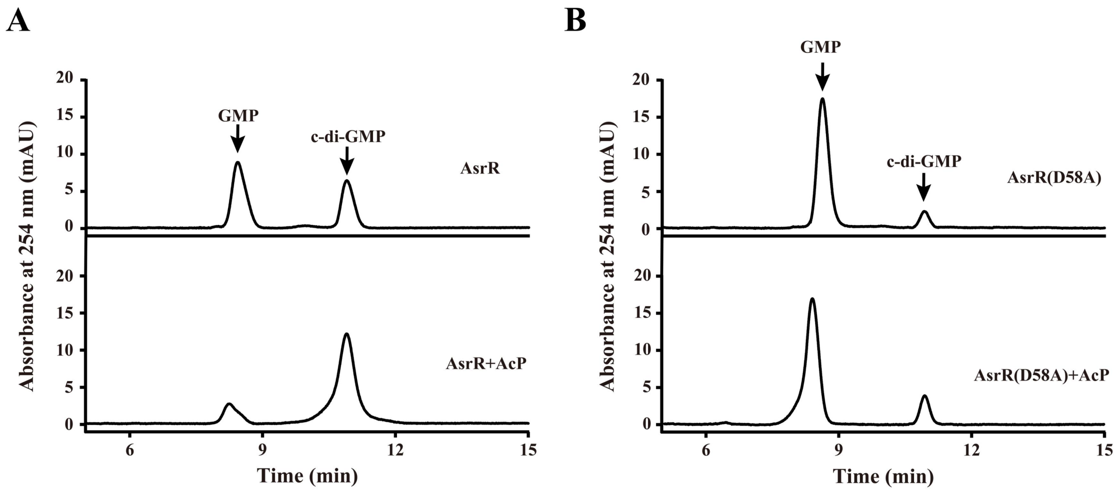

Fig. S3 Acetyl phosphate (AcP) treatment significantly reduces the PDE activity of wild-type AsrR, but not $AsrR_{D58A}$.

(A and B) The purified wild-type AsrR (A) and $AsrR_{D58A}$ (B) were pretreated by 25 mM AcP for 30 min at 30 °C before their reaction with c-di-GMP. Proteins without treatment were also used for reaction with c-di-GMP. The reaction products were analyzed by HPLC. HPLC spectra shown are representatives of three independent experiments with similar results.

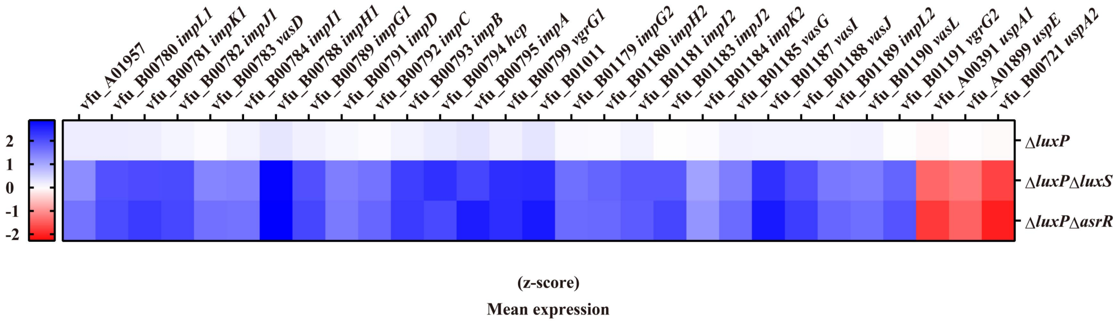

Fig. S4 The expression of several genes related to stress tolerance and protein secretion was showed by heatmap

Heatmap showing z-scores of normalized, $\log_2$-transformed expression of genes involved in stress tolerance and protein secretion in Δ*luxP*Δ*luxS* or Δ*luxP*Δ*asrR* compared to the Δ*luxP* in RNA-Seq data ($n = 3$ biological replicates).

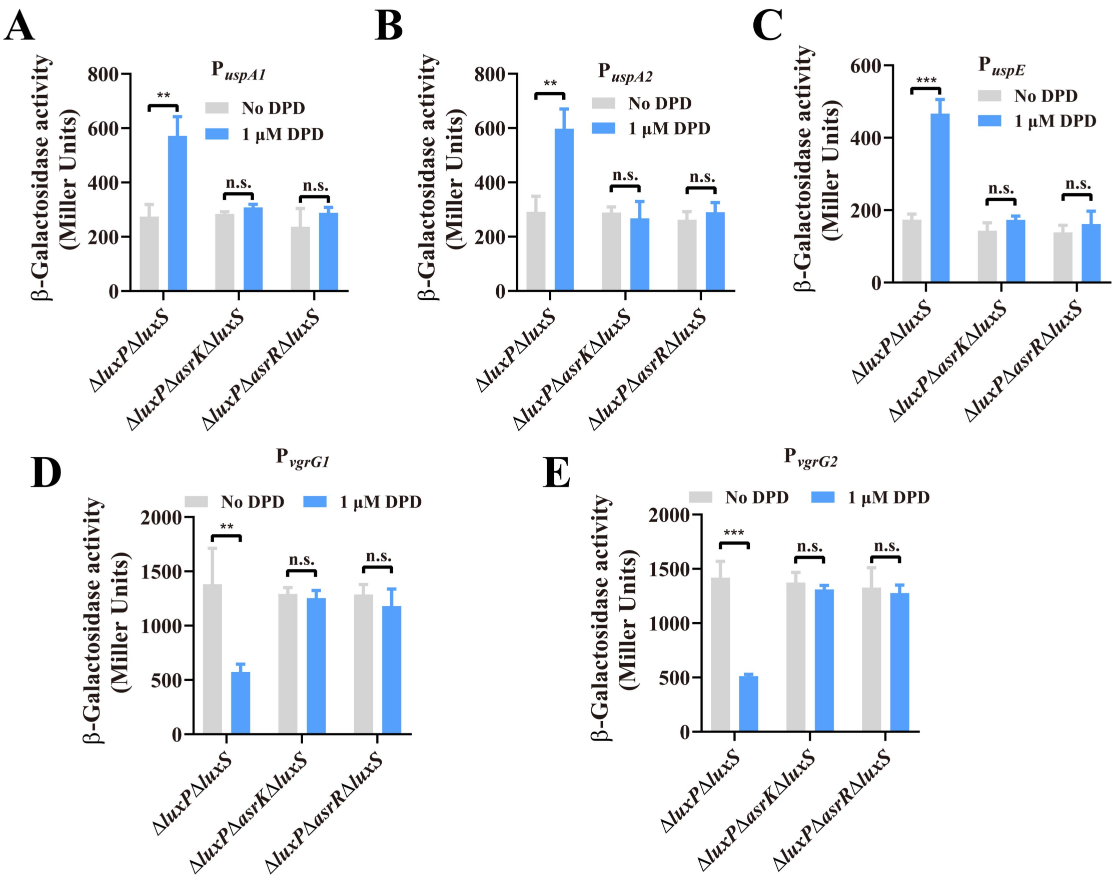

Fig. S5 The promoter activities of *uspA1*, *uspA2*, *uspE*, *vgrG1* and *vgrG2* in Δ*luxP*Δ*luxS* and its derivative mutants in the presence or absence of DPD

(A-E) Cells of Δ*luxP*Δ*luxS* and its derivative mutants cultured in LMB medium were supplied with or without 1 μM DPD at the time of inoculation and the promoter activities of *uspA1* (A), *uspA2* (B), *uspE* (C), *vgrG1* (D) and *vgrG2* (E) were monitored using the β-galactosidase activity assays. In A-E, statistical analyses were carried out by results from three independent experiments, each experiment having three technical replicates. Data are mean ± s.d. and the Student's *t*-test was used for these analyses. n.s., not significant; **, $P < 0.01$; ***, $P < 0.001$.

Table S1 List of strains and plasmids used in this study

Strains and plasmids	Relevant characteristics*	Source
**Strains**		
V. furnissii		
NCTC 11218	Wild-type	Laboratory stock
Δ*luxP*	*luxp* deletion mutant in NCTC 11218	This study
Δ*luxS*	*luxS* deletion mutant in NCTC 11218	This study
Δ*luxP*Δ*luxS*	*luxS* deletion mutant in Δ*luxP*	This study
Δ*luxP*Δ*asrK*	*asrK* deletion mutant in Δ*luxP*	This study
Δ*luxP*Δ*asrR*	*asrR* deletion mutant in Δ*luxP*	This study
Δ*luxP*Δ*uspA1*	*uspA1* deletion mutant in Δ*luxP*	This study
Δ*luxP*Δ*uspA2*	*uspA2* deletion mutant in Δ*luxP*	This study
Δ*luxP*Δ*uspE*	*uspE* deletion mutant in Δ*luxP*	This study
Δ*asrK*Δ*luxS*	*asrK* deletion mutant in Δ*luxS*	This study
asrR(58A)Δ*luxP*	*asrR*(58A) deletion mutant in Δ*luxP*Δ*asrR*	This study
asrR(58E)Δ*luxP*	*asrR*(58E) deletion mutant in Δ*luxP*Δ*asrR*	This study
Δ*luxP*Δ*uspA1*Δ*uspA2*Δ*uspE*	*uspA1*/*uspA2*/*uspE* deletion mutant in Δ*luxP*	This study
Δ*luxP*Δ*asrR*Δ*asrK*	*asrR*/*asrK* deletion mutant in Δ*luxP*	This study
Δ*luxP*Δ*asrK*Δ*luxS*	*asrK*/*luxS* deletion mutant in Δ*luxP*	This study
Δ*luxP*Δ*asrR*Δ*luxS*	*asrR*/*luxS* deletion mutant in Δ*luxP*	This study
Δ*luxP*Δ*uspA1*Δ*luxS*	*uspA1*/*luxS* deletion mutant in Δ*luxP*	This study
Δ*luxP*Δ*uspA2*Δ*luxS*	*uspA2*/*luxS* deletion mutant in Δ*luxP*	This study
Δ*luxP*Δ*uspE*Δ*luxS*	*uspE*/*luxS* deletion mutant in Δ*luxP*	This study
asrR(58A)Δ*luxP*Δ*luxS*	*asrR*(58A) deletion mutant in Δ*luxP*Δ*asrR*Δ*luxS*	This study
asrR(58E)Δ*luxP*Δ*luxS*	*asrR*(58E) deletion mutant in Δ*luxP*Δ*asrR*Δ*luxS*	This study
Δ*luxP*Δ*uspA1*Δ*uspA2*Δ*uspE*Δ*luxS*	*uspA1*/*uspA2*/*uspE*Δ*luxS* deletion mutant in Δ*luxP*	This study
Δ*luxP* (pBAD22-Cm)	Δ*luxP* containing pBAD22-Cm	This study
Δ*luxP* (pBAD22-Cm-*luxP*)	Δ*luxP* containing pBAD22-Cm-*luxP*	This study
Δ*luxS* (pBAD22-Cm)	Δ*luxS* containing pBAD22-Cm	This study
Δ*luxS* (pBAD22-Cm-*luxS*)	Δ*luxS* containing pBAD22-Cm-*luxS*	This study
Δ*luxP*Δ*luxS* (pBAD22-Cm)	Δ*luxP*Δ*luxS* containing pBAD22-Cm	This study
Δ*luxP*Δ*luxS* (pBAD22-Cm-*luxS*)	Δ*luxP*Δ*luxS* containing pBAD22-Cm-*luxS*	This study
Δ*luxP*Δ*asrK* (pBAD22-Cm)	Δ*luxP*Δ*asrK* containing pBAD22-Cm	This study
Δ*luxP*Δ*asrK* (pBAD22-Cm-*asrK*)	Δ*luxP*Δ*asrK* containing pBAD22-Cm-*asrK*	This study
Δ*luxP*Δ*asrR* (pBAD22-Cm)	Δ*luxP*Δ*asrR* containing pBAD22-Cm	This study
Δ*luxP*Δ*asrR* (pBAD22-Cm-*asrR*)	Δ*luxP*Δ*asrR* containing pBAD22-Cm-*asrR*	This study
Δ*luxP*Δ*uspA1* (pBAD22-Cm)	Δ*luxP*Δ*uspA1* containing pBAD22-Cm	This study
Δ*luxP*Δ*uspA1* (pBAD22-Cm-*uspA1*)	Δ*luxP*Δ*uspA1* containing pBAD22-Cm-*uspA1*	This study
Δ*luxP*Δ*uspA2* (pBAD22-Cm)	Δ*luxP*Δ*uspA2* containing pBAD22-Cm	This study
Δ*luxP*Δ*uspA2* (pBAD22-Cm-*uspA2*)	Δ*luxP*Δ*uspA2* containing pBAD22-Cm-*uspA2*	This study
Δ*luxP*Δ*uspE* (pBAD22-Cm)	Δ*luxP*Δ*uspE* containing pBAD22-Cm	This study
Δ*luxP*Δ*uspE* (pBAD22-Cm-*uspE*)	Δ*luxP*Δ*uspE* containing pBAD22-Cm-*uspE*	This study
Δ*luxP*Δ*uspA1*Δ*uspA2*Δ*uspE* (pBAD22-Cm)	Δ*luxP*Δ*uspA1*Δ*uspA2*Δ*uspE* containing pBAD22-Cm	This study
Δ*luxP*Δ*asrR*Δ*asrK* (pBAD22-Cm)	Δ*luxP*Δ*asrR*Δ*asrK* containing pBAD22-Cm	This study

Δ*luxP*Δ*asrK*Δ*luxS* (pBAD22-Cm)	Δ*luxP*Δ*asrK*Δ*luxS* containing pBAD22-Cm	This study
Δ*luxP*Δ*asrR*Δ*luxS* (pBAD22-Cm)	Δ*luxP*Δ*asrR*Δ*luxS* containing pBAD22-Cm	This study
Δ*luxP*Δ*uspA1*Δ*luxS* (pBAD22-Cm)	Δ*luxP*Δ*uspA1*Δ*luxS* containing pBAD22-Cm	This study
Δ*luxP*Δ*uspA2*Δ*luxS* (pBAD22-Cm)	Δ*luxP*Δ*uspA2*Δ*luxS* containing pBAD22-Cm	This study
Δ*luxP*Δ*uspE*Δ*luxS* (pBAD22-Cm)	Δ*luxP*Δ*uspE*Δ*luxS* containing pBAD22-Cm	This study
Δ*luxP*Δ*uspA1*Δ*uspA2*Δ*uspE*Δ*luxS* (pBAD22-Cm)	Δ*luxP*Δ*uspA1*Δ*uspA2*Δ*uspE*Δ*luxS* containing pBAD22-Cm	This study
Δ*luxP*-A1Z	Δ*luxP attB*::P_{uspA1}-*lacZ*	This study
Δ*luxP*Δ*luxS*-A1Z	Δ*luxP*Δ*luxS attB*::P_{uspA1}-*lacZ*	This study
Δ*luxP*Δ*asrK*-A1Z	Δ*luxP*Δ*asrK attB*::P_{uspA1}-*lacZ*	This study
Δ*luxP*Δ*asrR*-A1Z	Δ*luxP*Δ*asrR attB*::P_{uspA1}-*lacZ*	This study
Δ*luxP*Δ*asrK*Δ*luxS*-A1Z	Δ*luxP*Δ*asrK*Δ*luxS attB*::P_{uspA1}-*lacZ*	This study
Δ*luxP*Δ*asrR*Δ*luxS*-A1Z	Δ*luxP*Δ*asrR*Δ*luxS attB*::P_{uspA1}-*lacZ*	This study
Δ*luxP*-A2Z	Δ*luxP attB*::P_{uspA2}-*lacZ*	This study
Δ*luxP*Δ*luxS*-A2Z	Δ*luxP*Δ*luxS attB*::P_{uspA2}-*lacZ*	This study
Δ*luxP*Δ*asrK*-A2Z	Δ*luxP*Δ*asrK attB*::P_{uspA2}-*lacZ*	This study
Δ*luxP*Δ*asrR*-A2Z	Δ*luxP*Δ*asrR attB*::P_{uspA2}-*lacZ*	This study
Δ*luxP*Δ*asrK*Δ*luxS*-A2Z	Δ*luxP*Δ*asrK*Δ*luxS attB*::P_{uspA2}-*lacZ*	This study
Δ*luxP*Δ*asrR*Δ*luxS*-A2Z	Δ*luxP*Δ*asrR*Δ*luxS attB*::P_{uspA2}-*lacZ*	This study
Δ*luxP*-EZ	Δ*luxP attB*::P_{uspE}-*lacZ*	This study
Δ*luxP*Δ*luxS*-EZ	Δ*luxP*Δ*luxS attB*::P_{uspE}-*lacZ*	This study
Δ*luxP*Δ*asrK*-EZ	Δ*luxP*Δ*asrK attB*::P_{uspE}-*lacZ*	This study
Δ*luxP*Δ*asrR*-EZ	Δ*luxP*Δ*asrR attB*::P_{uspE}-*lacZ*	This study
Δ*luxP*Δ*asrK*Δ*luxS*-EZ	Δ*luxP*Δ*asrK*Δ*luxS attB*::P_{uspE}-*lacZ*	This study
Δ*luxP*Δ*asrR*Δ*luxS*-EZ	Δ*luxP*Δ*asrR*Δ*luxS attB*::P_{uspE}-*lacZ*	This study
Δ*luxP*-G1Z	Δ*luxP attB*::P_{vgrG1}-*lacZ*	This study
Δ*luxP*Δ*luxS*-G1Z	Δ*luxP*Δ*luxS attB*::P_{vgrG1}-*lacZ*	This study
Δ*luxP*Δ*asrK*-G1Z	Δ*luxP*Δ*asrK attB*::P_{vgrG1}-*lacZ*	This study
Δ*luxP*Δ*asrR*-G1Z	Δ*luxP*Δ*asrR attB*::P_{vgrG1}-*lacZ*	This study
Δ*luxP*Δ*asrK*Δ*luxS*-G1Z	Δ*luxP*Δ*asrK*Δ*luxS attB*::P_{vgrG1}-*lacZ*	This study
Δ*luxP*Δ*asrR*Δ*luxS*-G1Z	Δ*luxP*Δ*asrR*Δ*luxS attB*::P_{vgrG1}-*lacZ*	This study
Δ*luxP*-G2Z	Δ*luxP attB*::P_{vgrG2}-*lacZ*	This study
Δ*luxP*Δ*luxS*-G2Z	Δ*luxP*Δ*luxS attB*::P_{vgrG2}-*lacZ*	This study
Δ*luxP*Δ*asrK*-G2Z	Δ*luxP*Δ*asrK attB*::P_{vgrG2}-*lacZ*	This study
Δ*luxP*Δ*asrR*-G2Z	Δ*luxP*Δ*asrR attB*::P_{vgrG2}-*lacZ*	This study
Δ*luxP*Δ*asrK*Δ*luxS*-G2Z	Δ*luxP*Δ*asrK*Δ*luxS attB*::P_{vgrG2}-*lacZ*	This study
Δ*luxP*Δ*asrR*Δ*luxS*-G2Z	Δ*luxP*Δ*asrR*Δ*luxS attB*::P_{vgrG2}-*lacZ*	This study
E. coli		
BL21(DE3)	Host for expression vector pET-28a	Novagen
LuxS$^-$ BL21(DE3)	Δ*luxS* deletion mutant in *E. coli* BL21(DE3)	(3)
TG1	Host for cloning	Stratagene
S17-1λpir	λ-pir lysogen of S17-1, F$^-$ *thi pro hsdR* [RP4-2 Tc:: Mu Km::Tn7 (Tp Sm)]	(4)
V. harveyi		

V. harveyi MM32	*luxN*::*cat luxS*::Tn*5kan*	(5)
**Plasmids**		
pDM4	Cm^r; *sacB*-based gene replacement vector	(6)
pDM4-Δ*luxP*	Cm^r; Δ*luxP* in pDM4	This study
pDM4-Δ*luxS*	Cm^r; Δ*luxS* in pDM4	This study
pDM4-Δ*asrK*	Cm^r; Δ*asrK* in pDM4	This study
pDM4-Δ*asrR*	Cm^r; Δ*asrR* in pDM4	This study
pDM4-Δ*uspA1*	Cm^r; Δ*uspA1* in pDM4	This study
pDM4-Δ*uspA2*	Cm^r; Δ*uspA2* in pDM4	This study
pDM4-Δ*uspE*	Cm^r; Δ*uspE* in pDM4	This study
pDM4- *asrR*(D58A)	Cm^r; *asrR*(D58A) in pDM4	This study
pDM4- *asrR*(D58E)	Cm^r; *asrR*(D58E) in pDM4	This study
pBAD22	Amp^r; cloning vector containing araC	Laboratory stock
pBAD22-Cm	Cm^r; pBAD22 with the ampicillin resistance gene replaced by a chloramphenicol resistance gene	This study
pBAD22-Cm-*luxP*	*luxP* cloned into pBAD22-Cm for complementation	This study
pBAD22-Cm-*luxS*	*luxS* cloned into pBAD22-Cm for complementation	This study
pBAD22-Cm-*asrK*	*asrK* cloned into pBAD22-Cm for complementation	This study
pBAD22-Cm-*asrR*	*asrR* cloned into pBAD22-Cm for complementation	This study
pBAD22-Cm-*uspA1*	*uspA1* cloned into pBAD22-Cm for complementation	This study
pBAD22-Cm-*uspA2*	*uspA2* cloned into pBAD22-Cm for complementation	This study
pBAD22-Cm-*uspE*	*uspE* cloned into pBAD22-Cm for complementation	This study
pET-28a	Km^r; expression vector with N-terminal hexahistidine affinity tag	(7)
pET28a-*asrK-LBD*	pET-28a expressing *asrK-LBD*	This study
pET28a-*asrK-LBD*$_{Y215A}$	pET-28a expressing *asrK-LBD*$_{Y215A}$	This study
pET28a-*asrK-LBD*$_{D215A}$	pET-28a expressing *asrK-LBD*$_{D215A}$	This study
pET28a-*asrK-LBD*$_{D242A}$	pET-28a expressing *asrK-LBD*$_{D242A}$	This study
pET28a-*asrK*	pET-28a expressing *asrK*	This study
pET28a-*asrK*$_{Y213A}$	pET-28a expressing *asrK*$_{Y213A}$	This study
pET28a-*asrK*$_{D215A}$	pET-28a expressing *asrK*$_{D215A}$	This study
pET28a-*asrR*	pET-28a expressing *asrR*	This study
pET28a-*asrR*$_{D58A}$	pET-28a expressing *asrR*$_{D58A}$	This study
pET28a-*asrR*$_{D58E}$	pET-28a expressing *asrR*$_{D58E}$	This study
pDM4-*lacZ*	pDM4 derivative	(8)
uspA1-lacZ	751 bp upstream region of *uspA1* in pDM4-*lacZ*	This study
uspA2-lacZ	752 bp upstream region of *uspA2* in pDM4-*lacZ*	This study
uspE-lacZ	836 bp upstream region of *uspE* in pDM4-*lacZ*	This study
vgrG1-lacZ	898 bp upstream region of *vgrG1* in pDM4-*lacZ*	This study
vgrG2-lacZ	1086 bp upstream region of *vgrG2* in pDM4-*lacZ*	This study

***Km^r, Cm^r and Amp^r represent resistance to kanamycin, chloramphenicol and ampicillin, respectively.**

Table S2 List of primers used in this study

Name	Sequence (5′→3′)	
luxS Up F	AGTACGCGTCACTAGTGGGGCCCTTCTAGAAGCTTTGATGCGTTTCTTG	
luxS Up R	CAATCACGGTTTCAGCAATTGCGTTCATGCGAGTGT	to generate
luxS Low F	ACACTCGCATGAACGCAATTGCTGAAACCGTGATTG	pDM4-Δ*luxS*
luxS Low R	GAGAGCTCAGGTTACCCGCATGCAAGATCTATGTTGCGTCTGCGTGA	
luxS F	GGGCTAGCAGGAGGAATTCACCATGGTACCGATGCCATTATTAGACAGTTTTACC	to generate
luxS R	AAGCTTGCATGCCTGCAGGTCGACTCTAGATTAGTGCACTTTCAGCTCATTG	pBAD22-Cm-*luxS*
luxP Up F	AGTACGCGTCACTAGTGGGGCCCTTCTAGACAGCATCCACTGCCTTCA	
luxP Up R	GGTGGAACAGGCGTAAATAATAACTCTTCCAATCGCATTTC	to generate
luxP Low F	GAAATGCGATTGGAAGAGTTATTATTTACGCCTGTTCCACC	pDM4-Δ*luxP*
luxP Low R	GAGAGCTCAGGTTACCCGCATGCAAGATCTTGCCATCTTCCCATACCAT	
luxP F	GGGCTAGCAGGAGGAATTCACCATGGTACCGATGAAGAAAACGCTTTTTCTTTCTC	to generate
luxP R	AAGCTTGCATGCCTGCAGGTCGACTCTAGACTAACGGTCGGAATAACGAAAGG	pBAD22-Cm-*luxP*
asrK Up F	AGTACGCGTCACTAGTGGGGCCCTTCTAGATAACGAAACAGGCAACCAC	
asrK Up R	ATCCGTTTACAAATGGCGCAATACCGACTCCACCAAA	to generate
asrK Low F	TTTGGTGGAGTCGGTATTGCGCCATTTGTAAACGGAT	pDM4-Δ*asrK*
asrK Low R	GAGAGCTCAGGTTACCCGCATGCAAGATCTGGCTGACTGACTTTCACGAT	
asrK F	GGGCTAGCAGGAGGAATTCACCATGGTACCGATGAAATCAAACACCATCACGACAC	to generate
asrK R	AAGCTTGCATGCCTGCAGGTCGACTCTAGATTATCCCGTCGTTTCGTTGATG	pBAD22-Cm-*asrK*
vfu_A00028-LBD F	TCGAGGATCCATCATCATTGCCATCGTC	to generate pET28a-
vfu_ A00028-LBD R	GACTAAGCTTTCAAGAGGTAAAGGTGATTTG	*vfu_A00028-LBD*
vfu_A00298-LBD F	TCGAGGATCCCAGCGTGAAGTGATTGTC	to generate pET28a-
vfu_ A00298-LBD R	GACTAAGCTTTCAAATGCTTTTGCTCTCATG	*vfu_A00298-LBD*
vfu_A01158-LBD F	TCGAAGATCTTCTATTCAACAACTGAGC	to generate pET28a-
vfu_ A01158-LBD R	GACTAAGCTTTCACCCAATCACGGTGTAGAG	*vfu_A01158-LBD*
vfu_A01762-LBD F	TCGAGGAT*CC*ACGTGGTTGTCTGCCGATC	to generate pET28a-
vfu_ A01762-LBD R	GACTAAGCTTTCACGATGCAAACTCAGTGTC	*vfu_A01762-LBD*
vfu_B00142-LBD F	TCGAAGATCTTTGCTGCTGGTTACCGTC	to generate pET28a-
vfu_ B00142-LBD R	GACTAAGCTTTCACAACACGGCAAACCAAAG	*vfu_B00142-LBD*
vfu_B00258-LBD F	TGGGTCGCGGATCCGAATTCAGCGTCGATAACATGCGT	to generate pET28a-
vfu_B00258-LBD R	TCGAGTGCGGCCGCAAGCTTTTAGCGTTGGTATTTGTGTTCG	*vfu_B00258-LBD*
vfu_B00627-LBD F	TCGAGGAT*CC*CTGGGCTGGTTAGTGTAC	to generate pET28a-
vfu_B00627-LBD R	GACTAAGCTTTCATTCACTCAAGAGCACCCATTTC	*vfu_B00627-LBD*

vfu_B00963-LBD F	TCGAGGATCCGCACTGACCGCCATCGCATTG	to generate pET28a-*vfu_B00963-LBD*
vfu_B00963-LBD R	GACTAAGCTTTCAATTTTTGACCATCACGTG	
vfu_B01032-LBD F	TCGAGGATCCGCGTCGTTGAGTCGGTTG	to generate pET28a-*vfu_B01032-LBD*
vfu_B01032-LBD R	GACTAAGCTTTCACACATTGAACAAAATGGTG	
vfu_B00258-LBD$_{\text{D215A}}$ Up F	TGACAGATCTTATATCAATGAGCAAGTGAACAACGAAC	to generate pET28a-*vfu_B00258-LBD*$_{\text{D215A}}$
vfu_B00258-LBD$_{\text{D215A}}$ Up R	ACCGGCGGGAGCAATGTACACATCCGTCCACACCCCCGTTTTTTG	
vfu_B00258-LBD$_{\text{D215A}}$ Low F	GTGTACATTGCTCCCGCCGGTTTGGGCTGGATGGCATCGTC	
vfu_B00258-LBD$_{\text{D215A}}$ Low R	TGACAAGCTTTCAAATCATCAACAGTTGCCATTTCGTTTC	
vfu_B00258-LBD$_{\text{Y213A}}$ Up F	GCAAATGGGTCGCGGATCCGAATTCTATATCAATGAGCAAGTGAACAACG	to generate pET28a-*vfu_B00258-LBD*$_{\text{Y213A}}$
vfu_B00258-LBD$_{\text{Y213A}}$ Up R	GGGAGCAATGGCCACATCCGTCCACACCCC	
vfu_B00258-LBD$_{\text{Y213A}}$ Low F	GACGGATGTGGCCATTGCTCCCGCCGGTTT	
vfu_B00258-LBD$_{\text{Y213A}}$ Low R	GGTGCTCGAGTGCGGCCGCAAGCTTTCAAATCATCAACAGTTGCCATTTCGTTTC	
vfu_B00258-LBD$_{\text{D242A}}$ Up F	GCAAATGGGTCGCGGATCCGAATTCTATATCAATGAGCAAGTGAACAACG	to generate pET28a-*vfu_B00258-LBD*$_{\text{D242A}}$
vfu_B00258-LBD$_{\text{D215A}}$ Up R	TCACGGTGATAGCCAGCCCGACCACGCCTTCC	
vfu_B00258-LBD$_{\text{D242A}}$ Low F	GTCGGGCTGGCTATCACCGTGAG	
vfu_B00258-LBD$_{\text{D242A}}$ Low R	GGTGCTCGAGTGCGGCCGCAAGCTTTCAAATCATCAACAGTTGCCATTTCGTTTC	
full-length *asrK* F	GCAAATGGGTCGCGGATCCGAATTCATGAAATCAAACACCATCACGACACAAG	to generate pET28a-*asrK*
full-length *asrK* R	GGTGCTCGAGTGCGGCCGCAAGCTTTTATCCCGTCGTTTCGTTGATGCGCTCC	
asrK$_{\text{D215A}}$ Up F	GCAAATGGGTCGCGGATCCGAATTCATGAAATCAAACACCATCACGACACAAG	to generate pET28a-*asrK*$_{\text{D215A}}$
asrK$_{\text{D215A}}$ Up R	ACCGGCGGGAGCAATGTACACATCCGTCCACACCCCCGTTTTTTG	
asrK$_{\text{D215A}}$ Low F	GTGTACATTGCTCCCGCCGGTTTGGGCTGGATGGCATCGTC	
asrK$_{\text{D215A}}$ Low R	GGTGCTCGAGTGCGGCCGCAAGCTTTTATCCCGTCGTTTCGTTGATGCGCTCC	
AsrK$_{\text{Y213A}}$ Up F	GCAAATGGGTCGCGGATCCGAATTCATGAAATCAAACACCATCACGACACAAG	to generate pET28a-*asrK*$_{\text{Y213A}}$
asrK$_{\text{Y213A}}$ Up R	GGGAGCAATGGCCACATCCGTCCACACCCC	
asrK$_{\text{Y213A}}$ Low F	GACGGATGTGGCCATTGCTCCCGCCGGTTT	
asrK$_{\text{Y213A}}$ Low R	GGTGCTCGAGTGCGGCCGCAAGCTTTTATCCCGTCGTTTCGTTGATGCGCTCC	

asrR Up F	AGTACGCGTCACTAGTGGGGCCCTTCTAGAGCACTCTTTAGCCATATGTT	
asrR Up R	TATCGTTTCCTGCCAGTCTATTCCTCCTTGAGCAGTT	to generate
asrR Low F	AACTGCTCAAGGAGGAATAGACTGGCAGGAAACGATA	pDM4-Δ*asrR*
asrR Low R	GAGAGCTCAGGTTACCCGCATGCAAGATCTTGTAGGAGTCCCAACTATTG	
asrR F	GGGCTAGCAGGAGGAATTCACCATGGTACCGATGTTTGATGTGTCGTCGTCT	to generate
asrR R	AAGCTTGCATGCCTGCAGGTCGACTCTAGACTAATTGGCATAGCGAAGC	pBAD22-Cm-*asrR*
asrR$_{\mathrm{WT}}$ F	GGACAGCAAATGGGTCGCGGATCCGAATTCATGTTTGATGTGTCGTCGTCT	to generate
asrR$_{\mathrm{WT}}$ R	GTGGTGGTGCTCGAGTGCGGCCGCAAGCTTCTAATTGGCATAGCGAAGC	pET28a-*asrR*$_{\mathrm{WT}}$
asrR$_{\mathrm{D58A}}$ Up F	GGACAGCAAATGGGTCGCGGATCCGAATTCATGTTTGATGTGTCGTCGTCT	
asrR$_{\mathrm{D58A}}$ Up R	CATATCCGGCATCATGATAGCGAGCAGAATGAGATCA	to generate
asrR$_{\mathrm{D58A}}$ Low F	TGATCTCATTCTGCTCGCTATCATGATGCCGGATATG	pET28a-*asrR*$_{\mathrm{D58E}}$
asrR$_{\mathrm{D58A}}$ Low R	GTGGTGGTGCTCGAGTGCGGCCGCAAGCTTCTAATTGGCATAGCGAAGC	
asrR$_{\mathrm{D58E}}$ Up F	GGACAGCAAATGGGTCGCGGATCCGAATTCATGTTTGATGTGTCGTCGTCT	
asrR$_{\mathrm{D58E}}$ Up R	CATATCCGGCATCATGATTTCGAGCAGAATGAGATC	to generate
asrR$_{\mathrm{D58E}}$ Low F	GATCTCATTCTGCTCGAAATCATGATGCCGGATATG	pET28a-*asrR*$_{\mathrm{D58E}}$
asrR$_{\mathrm{D58E}}$ Low R	GTGGTGGTGCTCGAGTGCGGCCGCAAGCTTCTAATTGGCATAGCGAAGC	
asrR(D58A) Up F	AGTACGCGTCACTAGTGGGGCCCTTCTAGAGGGCTTGGTGCGTTGGTG	
asrR(D58A) Up R	CATCATGATAGCGAGCAGAATGAGATCAG	to generate pDM4-
asrR(D58A) Low F	ATTCTGCTCGCTATCATGATGCCGGATATG	*asrR*(D58A)
asrR(D58A) Low R	GAGAGCTCAGGTTACCCGCATGCAAGATCTGCGGGTACTGCGCCAAGG	
asrR(D58E) Up F	AGTACGCGTCACTAGTGGGGCCCTTCTAGAGGGCTTGGTGCGTTGGTG	
asrR(D58E) Up R	GCATCATGATCTCGAGCAGAATGAGATCAG	to generate pDM4-
asrR(D58E) Low F	ATTCTGCTCGAGATCATGATGCCGGATATG	*asrR*(D58E)
asrR(D58E) Low R	GAGAGCTCAGGTTACCCGCATGCAAGATCTGCGGGTACTGCGCCAAGG	
uspA1 Up F	AGCTTCTAGAGCTAGCATCGAGGGTTCC	
uspA1 Up R	TGAGCAATTAATGAGCTGGCGGGTTGAG	to generate
uspA1 LowF	GCCAGCTCATTAATTGCTCACCTGTCGACC	pDM4-Δ*uspA1*
uspA1 LowR	AGCTAGATCTATGCCGCCAATTGCCATC	
uspA1 F	AGCTGGTACCGCAAGGAGATAGCATTATGAG	to generate
uspA1 R	AGCTTCTAGATCTTCGTCGTCAGTCGTTG	pBAD22-Cm-*uspA1*

P*uspA1* F	AGCTGTCGACATACTTCGCCTAAGCTTG	To generate *uspA1-lacZ*
P*uspA1* R	AGCTTCTAGAACCGCACACCACTAAATC	
qRT *uspA1* F	TGGAAGCGTCTCAGAAACAG	for qRT-PCR
qRT *uspA1* R	GCACAGCTCGTTACTCAAATC	experiment
uspA2 Up F	AGCTTCTAGATGCAATGCGCTCGTCGTC	
uspA2 Up R	ACCGCAAATCAAACGACACCGCTTTATC	to generate
uspA2 LowF	GGTGTCGTTTGATTTGCGGTCATCACCATG	pDM4-Δ*uspA2*
uspA2 LowR	AGCTAGATCTCGTGCAGAAAAAGCGTCG	
uspA2 F	AGCTGGTACCGGTCGTGATGAAATACCAAC	to generate
uspA2 R	AGCTTCTAGATCAGATCGGAACCACCAG	pBAD22-Cm-*uspA2*
P*uspA2* F	AGCTGTCGACAAACCCATCAAACGACTC	To generate *uspA2-lacZ*
P*uspA2* R	AGCTTCTAGATCAAAACCGTTGTCGATG	
qRT *uspA2* F	AGAGTTGATCGACATTCAGGC	for qRT-PCR
qRT *uspA2* R	AGTTTGTCACTCAGATCGCC	experiment
uspE Up F	AGCTTCTAGAGTGTGTGAGATTCCATAC	
uspE Up R	CATCAATACCATCACCGACTCATCAACATAAG	to generate
uspE LowF	AGTCGGTGATGGTATTGATGAAGCACAAA	pDM4-Δ*uspE*
uspE LowR	AGCTAGATCTGAACACAGTGAACACGTC	
uspE F	AGCTGGTACCGATGAGTATCTATAGCAAAATC	to generate
uspE R	AGCTTCTAGATTAGCTGGCTGTTTTGGG	pBAD22-Cm-*uspE*
P*uspE* F	AGCTGTCGACAGATTCCATACGAAATTC	To generate *uspE-lacZ*
P*uspE* R	AGCTTCTAGAAATACTTCGCCGATGATC	
qRT *uspE* F	ATGTGTTGGAGTCGGTGATC	for qRT-PCR
qRT *uspE* R	TCAACTTCTGAGCCGACATG	experiment
P*vgrG1* F	AGCTCTCGAGAACCGCATCAGTGAAGAC	To generate *vgrG1-lacZ*
P*vgrG1* R	AGCTTCTAGAGTTGCGACTGAGCCTGTG	
qRT *vgrG1* F	AGTACACCGTCAACAGCATG	for qRT-PCR
qRT *vgrG1* R	ACTCAAAATCCGACTCACCG	experiment
P*vgrG2* F	AGCTGTCGACGTGACGGGTTGTGTTTTG	To generate *vgrG2-lacZ*
P*vgrG2* R	AGCTTCTAGAGTTTACGCGTTGAACCAG	
qRT *vgrG2* F	GTGGTAGATAAAGTCGCGGAG	for qRT-PCR
qRT *vgrG2* R	CTCTAAAGCAGGGACTAACGTG	experiment
16s F	GTAATGTCGGGAACTCCAGGG	for qRT-PCR
16s R	ACTCCAATCCGGATTACGACG	experiment

Supplementary References

1. Gavira JA, Gumerov VM, Rico-Jimenez M, Petukh M, Upadhyay AA, Ortega A, Matilla MA, Zhulin IB, Krell T. 2020. How bacterial chemoreceptors evolve novel ligand specificities. mBio 11:e03066-19.
2. Rico-Jimenez M, Munoz-Martinez F, Garcia-Fontana C, Fernandez M, Morel B, Ortega A, Ramos JL, Krell T. 2013. Paralogous chemoreceptors mediate chemotaxis towards protein amino acids and the non-protein amino acid gamma-aminobutyrate (GABA). Mol Microbiol 88:1230-1243.
3. Zhang L, Li S, Liu X, Wang Z, Jiang M, Wang R, Xie L, Liu Q, Xie X, Shang D, Li M, Wei Z, Wang Y, Fan C, Luo ZQ, Shen X. 2020. Sensing of autoinducer-2 by functionally distinct receptors in prokaryotes. Nat Commun 11:5371.
4. Lin J, Zhang W, Cheng J, Yang X, Zhu K, Wang Y, Wei G, Qian PY, Luo ZQ, Shen X. 2017. A *Pseudomonas* T6SS effector recruits PQS-containing outer membrane vesicles for iron acquisition. Nature Communication 8:14888.
5. Miller ST, Xavier KB, Campagna SR, Taga ME, Semmelhack MF, Bassler BL, Hughson FM. 2004. *Salmonella typhimurium* recognizes a chemically distinct form of the bacterial quorum-sensing signal AI-2. Mol Cell 15:677-687.
6. Zhao Y, Shao F. 2015. The NAIP-NLRC4 inflammasome in innate immune detection of bacterial flagellin and type III secretion apparatus. Immunol Rev 265:85-102.
7. Wang Z, Xie X, Shang D, Xie L, Hua Y, Song L, Yang Y, Wang Y, Shen X, Zhang L. 2022. A c-di-GMP signaling cascade controls motility, biofilm formation, and virulence in *Burkholderia thailandensis*. Appl Environ Microbiol 88:e0252921.
8. Li S, Sun H, Li J, Zhao Y, Wang R, Xu L, Duan C, Li J, Wang Z, Liu Q, Wang Y, Ouyang S, Shen X, Zhang L. 2022. Autoinducer-2 and bile salts induce c-di-GMP synthesis to repress the T3SS via a T3SS chaperone. Nat Commun 13:6684.
